# Synergistic Interactions between the Molecular and Neuronal Circadian Networks Drive Robust Behavioral Circadian Rhythms in *Drosophila melanogaster*

**DOI:** 10.1371/journal.pgen.1004252

**Published:** 2014-04-03

**Authors:** Ron Weiss, Osnat Bartok, Shaul Mezan, Yuval Malka, Sebastian Kadener

**Affiliations:** Biological Chemistry Department, Silberman Institute of Life Sciences, The Hebrew University of Jerusalem, Jerusalem, Israel; University of Pennsylvania, United States of America

## Abstract

Most organisms use 24-hr circadian clocks to keep temporal order and anticipate daily environmental changes. In *Drosophila melanogaster* CLOCK (CLK) and CYCLE (CYC) initiates the circadian system by promoting rhythmic transcription of hundreds of genes. However, it is still not clear whether high amplitude transcriptional oscillations are essential for circadian timekeeping. In order to address this issue, we generated flies in which the amplitude of CLK-driven transcription can be reduced partially (approx. 60%) or strongly (90%) without affecting the average levels of CLK-target genes. The impaired transcriptional oscillations lead to low amplitude protein oscillations that were not sufficient to drive outputs of peripheral oscillators. However, circadian rhythms in locomotor activity were resistant to partial reduction in transcriptional and protein oscillations. We found that the resilience of the brain oscillator is depending on the neuronal communication among circadian neurons in the brain. Indeed, the capacity of the brain oscillator to overcome low amplitude transcriptional oscillations depends on the action of the neuropeptide PDF and on the *pdf*-expressing cells having equal or higher amplitude of molecular rhythms than the rest of the circadian neuronal groups in the fly brain. Therefore, our work reveals the importance of high amplitude transcriptional oscillations for cell-autonomous circadian timekeeping. Moreover, we demonstrate that the circadian neuronal network is an essential buffering system that protects against changes in circadian transcription in the brain.

## Author Summary

Circadian clocks allow organisms to predict daily environmental changes. These clocks time the sleep/wake cycles and many other physiological and cellular pathways to 24hs rhythms. The current model states that circadian clocks keep time by the use of biochemical feedback loops. These feedback loops are responsible for the generation of high amplitude oscillations in gene expression. Abolishment of circadian transcriptional oscillations has been shown to abolish circadian function. Previous studies addressing this issue utilize manipulations in which the abolishment of the transcriptional oscillations is very dramatic and involves strong up or down-regulation of circadian genes. In this study we generated fruit flies in which we diminished the amplitude of circadian oscillations in a controlled way. We found that a decrease of more than 50% in the amplitude of circadian oscillations leads to impaired function of circadian physiological outputs in the periphery but does not significantly affect circadian behavior. This suggests that the clock in the brain has a specific compensatory mechanism. Moreover, we found that flies with reduced oscillation and impaired circadian neuronal communication display aberrant circadian rhythms. These finding support the idea of network buffering mechanisms that allows the brain to produce circadian rhythms even with low amplitude molecular oscillations.

## Introduction

Most organisms use 24-hr circadian clocks to keep temporal order and anticipate daily environmental changes. These clocks are based on self-sustained biochemical oscillators that manifest at the molecular, physiological and behavioral levels [for review see [1], [2]]. Circadian clocks have been proposed to work on cell-autonomous basis and to be generated by interconnected complex transcriptional-posttranslational feedback loops [1].

In *Drosophila melanogaster*, the master genes *Clock* (*Clk*) and *cycle* (*cyc*) activate the circadian system by promoting rhythmic transcription of several key genes. Three of these target gene products, PERIOD (PER) [3], TIMELESS (TIM) [4], and CWO [5]–[7] repress CLK-CYC mediated transcription on a daily basis. The CLK-CYC heterodimer also activates the expression of VRI and PDP1, which are responsible for the oscillation of *Clk* mRNA [8], [9]. Post-transcriptional and post-translational regulation also contributes to circadian timekeeping [10]–[12]. A central role for transcriptional feedback loops has been challenged by the idea that other modes of regulation, like phosphorylation of key clock proteins as PER are more important for circadian timekeeping. However, other work has re-confirmed the importance of transcriptional regulation for timekeeping in *Drosophila* and mammals [13]–[15].

Oscillations of clock gene products occur in variety of fly tissues [16]. However, discrete circadian pacemaker neurons in the brain are responsible for the generation of locomotor activity rhythms [17]. These brain pacemakers show robust oscillations at the molecular level even after weeks in constant darkness (DD) [18]. Approximately 150 neurons drive circadian locomotor activity rhythms. They have been divided into several subgroups based on their location and expression of the clock genes PER, TIM, and CRY and the neuropeptide PDF [19], [20]. These groups are called the ventral lateral (sLNvs and lLNvs), dorsal lateral (LNds), and dorsal (DN1s, DN2s, and DN3s) neurons. The neuropeptide PDF, which is expressed exclusively in the LNvs, is essential for normal circadian patterns of activity in LD and persistent circadian rhythms in DD [17], [21]–[23]. Recent evidence suggests that PDF synchronizes the brain circadian neurons [18], [24]–[30].

Peripheral clocks are spread throughout the fly body and regulate a plethora of functions that include eclosion, olfaction, detoxification, and immunity [1]. These clocks have strong molecular rhythms in light/dark (LD) conditions. Although these peripheral clock rhythms disappear in DD in most tissues [31], a few peripheral oscillators perform well in DD. This may be due to stronger or non-dampening transcriptional oscillations (e.g., olfaction [32]) or signaling from the brain oscillator (i.e., eclosion rhythms [33]).

Although both types of oscillators are thought to work in a cell-autonomous fashion, the neurons in the brain central oscillator communicate timing information to each other. This communication was proposed to be responsible for synchronized molecular oscillations in individual cells, which leads to robust behavioral rhythms in DD [18], [24]–[27]. Conversely, in mammals, communication between circadian neurons provides robustness to the brain oscillator [2], [34], [35]–[38]. Despite the great advances achieved in the last few years, the relative importance of intra and intercellular contributions to generation of robust circadian behavioral is still not well understood.

A few years ago, we generated flies carrying the UAS-*ClkGR* transgene [5]. This transgene encodes a fusion protein (CLKGR) between the full *Drosophila* CLK protein and the ligand binding domain of rat glucocorticoid receptor (GR). This type of fusion is widely used for generating inducible systems, as the presence of the GR ligand binding domain assures cytoplasmic retention, which can be reversed by addition of GR ligands (like the artificial analog Dexamethasone [39]). Indeed, we previously demonstrated that addition of Dexamethasone to fly tissues expressing the CLKGR fusion leads to quick and very strong induction of CLK-driven transcription [5]. Importantly, addition of the inducer has no other effects, as there is no endogenous glucocorticoid-like receptor or ligand in flies.

Here, we generated flies, herein referred to as TIM-CLKGR flies that express this fusion protein in *tim*-expressing cells (*tim-gal4*/+; UAS-*ClkGR/+*). Surprisingly, expression of CLKGR in a wild type background without adding dexamethasone reduced the amplitude of CLK-driven circadian transcriptional oscillations by more than 50%. This resulted in low-amplitude protein oscillation, and impaired activity of peripheral circadian oscillators. TIM-CLKGR flies displayed almost no transcriptional oscillations of peripheral organ genes and had aberrant eclosion rhythms and sleep disturbances. Interestingly, locomotor activity rhythms were only weakly affected in TIM-CLKGR flies, demonstrating that the brain circadian clock is more resilient to changes in transcriptional amplitude than peripheral clocks. The resilience of the central oscillator is dependent on an intact and functioning circadian neuronal network structure. Indeed, flies in which the *pdf* neuropeptide pathway is impaired (by mutations in *pdf* or the in the PDF receptor *Han*) showed very strong behavioral phenotypes upon expression of the CLKGR protein. In agreement with the prominent role of the *pdf* signaling pathway, we found that the *pdf*-expressing cells have a key role in buffering the adverse effects of low amplitude circadian oscillations in the brain. In sum, our results provide strong evidence that high amplitude circadian oscillations in combination with intact neuronal structure are key constituents of robust circadian systems.

## Results

### Expression of CLK-GR impairs transcriptional oscillations *in vivo*

**Figure 1:**
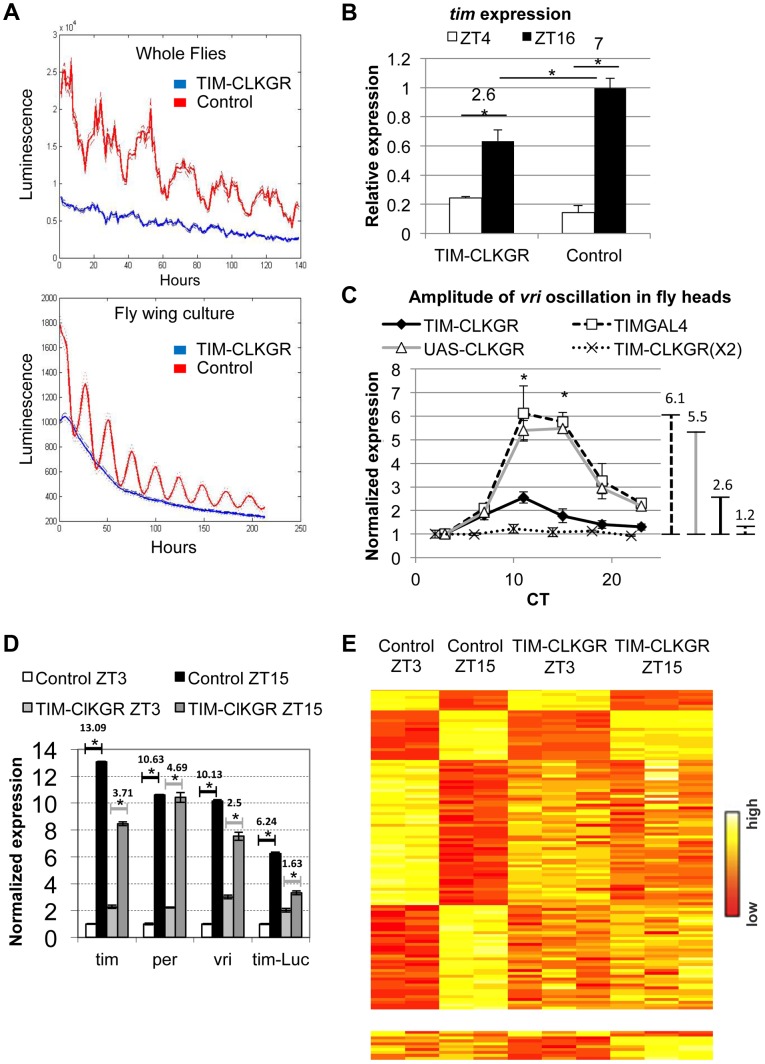
CLKGR expression decreases the amplitude of CLK-driven transcriptional oscillations. **A.** Molecular oscillations assessed by the *tim*-luciferase transgene are abolished in TIM-CLKGR flies. The plots show the mean data (N = 24, up whole flies, down isolated fly wings). The dotted lines indicate the SEM. Blue line refers to TIM-CLKGR and red line to control (UAS-*ClkGR/+*). Experiments were performed in 12∶12 Light∶Dark cycles (LD) **B.** TIM-CLKGR flies display low amplitude *tim* mRNA oscillations in LD. We assayed the levels of *tim* mRNA in fly heads collected at two different time points by Real-Time PCR. We represented it as signal over control mRNA (using the housekeeping gene *actin*). Plot values are average of three biological repeats for TIM-CLKGR and two biological repeats for control (each biological replica was measured by triplicate). We utilized UAS-*ClkGR/+* flies as control in this experiment. Error bars represent SEM. Numbers on horizontal lines represent the ratio between ZT16 and ZT4. T-test was performed to determine statistical significance. *p<0.05. **C.** TIM-CLKGR flies display low amplitude *vri* mRNA oscillations during the first day in constant darkness (DD1). Levels of *vri* were assayed by Real-Time PCR from fly heads collected at 6 different timepoints during DD1. We tested flies with one copy of the UAS-*ClkGR* transgene (TIM-CLKGR) and flies with two copies of the UAS-*ClkGR* transgene (TIM-CLKGR(X2)). As control we used the fly strains, *tim-gal4/+* (TIMGAL4) and UAS-*ClkGR/+* (UAS-CLKGR). The levels are normalized to two housekeeping genes (Rp49 and RpS18) and were performed from three biological replicates. For each measurement we performed three technical replicas. In order to appreciate differences in amplitude, we normalized the relative expression to the first time-point. Error bars represent SEM. Lines in the right side of the chart show the amplitudes size. One-way Anova was performed to determine statistical significance of the differences between TIM-CLKGR, TIMGAL4 and UAS-CLKGR flies. *p<0.05. **D.** CLKGR expression diminishes amplitude of circadian mRNA oscillations. The graph shows the mean of expression for *tim*, *per*, *vri* and *luciferase* mRNAs (that is expressed under *tim* promoter) in control (UAS-*ClkGR/+*) and TIM-CLKGR flies. Gene expression was measured from fly heads collected at ZT3 and ZT15. We collected three samples of TIM-CLKGR and two samples of control for each time point. Flies were held in LD (12∶12). Levels of expression were assessed by oligonucleotide microarrays and are normalized to the time-point displaying lower expression in control flies. Horizontals lines show the fold change between time points. Error bars represent SEM. T-test was performed to determine statistical significance between time points. *p<0.05. **E.** CLKGR expression leads to global changes in amplitude of circadian oscillations. Heat plots for the microarray experiment described in D. Upper chart displays 110 genes that show differential expression between timepoints in control flies (t-test *p*<0.05, 2 fold threshold), lower chart displays genes that show differential expression between timepoints only in TIM-CLKGR flies.

We generated flies expressing the *CLKGR* transgene [5] under the control of the *tim-gal4* driver in a wild-type *Clk* background; hence these flies (TIM-CLKGR flies) carry two endogenous wild-type alleles of *Clk* and the UAS-*ClkGR* transgene. These flies also contain a *tim*-*luciferase* transgene (*tim-luc*), which allows *in vivo* monitoring of CLK-CYC mediated transcription [40]. Control whole flies displayed strong transcriptional rhythms, as did isolated wings (Figure 1A, red line). Surprisingly, we failed to detect luciferase oscillations in the absence of dexamethasone in TIM-CLKGR whole flies and isolated wings (Figure 1A, blue line). This suggests that the CLKGR fusion protein interferes with the endogenous molecular clock. In order to rule out the possibility that the lack of oscillations is due to toxic effect of CLKGR protein expression on the survival of the tissue, we evaluated the levels of the luciferase expression after adding dexamethasone to the culture media. Indeed, addition of dexamethasone resulted in a significant increase in the luciferase levels, demonstrating that CLKGR expression impairs the circadian system rather than affects the survival of the tissue (Figure S1).

To determine whether this fusion protein also interfered with the oscillation of endogenous CLK-driven mRNAs, we performed Real-Time RT PCR to assess *tim* mRNA levels from fly heads of control and TIM-CLKGR flies collected at two time points. Figure 1B shows that expression of CLKGR in *tim*-expressing cells significantly diminished the oscillation amplitude of *tim* mRNA in Light∶Dark (LD) conditions (Figure 1B). Interestingly, the effect is mainly restricted to the amplitude of oscillations rather than to the average levels of *tim* mRNA (see below). The differences in levels observed between *tim* mRNA levels and the *tim-luciferase* reporter are likely due to the fact that in the *tim-luciferase* transgene transcription is driven by only 760 bases of the *tim* promoter (compared to the 7 Kb genomic fragment necessary to fully recapitulate *tim* gene expression in time and space, Shaul Mezan personal communication). Therefore, we conclude that CLKGR interferes with endogenous CLK activity, and that this causes low amplitude transcriptional oscillations without much effect in the total levels of overall CLK-driven transcription.

We then analyzed the effect of the CLKGR protein on transcriptional oscillations in constant darkness (DD). We performed RT-PCR on total RNA from control and TIM-CLKGR fly heads collected at different circadian time points (CT). The amplitude of *vri* mRNA oscillations was severely diminished (less than 50% of controls, 2.6 fold difference across the day instead of 5.5 and 6.1 of the control strains) in TIM-CLKGR flies compared to the control flies (Figure 1C). Interestingly, we found that the main consequence of expression of CLKGR is at the level of amplitude, as overall *vri* levels are not significantly different between control and TIM-CLKGR flies (Figure S2A). We also performed a similar assessment in flies carrying two copies of the UAS-*ClkGR* transgene (TIM-CLKGR(X2) flies). Interestingly, we found that *vri* mRNA oscillations were almost abolished in these flies (Figure 1C). These results suggest that the CLKGR fusion protein acts as a weak dominant negative regulator of transcriptional oscillations.

In order to evaluate genome-wide effects of the expression of CLKGR, we collected fly heads from control and TIM-CLKGR at two different time points in LD (ZT3 and ZT15) and analyzed their transcriptome using oligonucleotides microarrays. In agreement with the RT-PCR analyses, *vri* mRNA showed significantly decreased amplitudes of oscillation without much effect in the total mRNA levels (Figure 1D and S2B). We observed a similar decrease for *per*, *tim*, *Pdp1*, *cwo* and the *luciferase* mRNAs from the *tim-luciferase* reporter which is also present in this strain (Figure 1D). In agreement with what we determined by RT-PCR for *vri* (Figure S2A), expression of the CLKGR transgene does not significantly affect the overall levels of the core CLK-transcriptional targets in this dataset (*per*, *tim*, *Pdp1* and *cwo*, see Figure S2B). Interestingly, expression of CLKGR significantly decreased the number of probes differentially expressed between the two assayed time points (the number of oscillating genes is less than one third in TIM-CLKGR flies compare to control). Moreover, more than half (17/24) of the mRNAs that still show differential expression between the two timepoints in TIM-CLKGR flies did so with diminished amplitude with respect to control flies. This was the case for both direct as well as indirect (e.g., *cry*) CLK-transcriptional targets (Figure 1E; Dataset S1). Therefore, we conclude that expression of the CLKGR fusion interferes with the endogenous CLK by an unknown mechanism. In any case this transgene allows us to study the consequences of diminishing CLK-driven transcriptional oscillations by approx. 60% or 90% (by using TIM-CLKGR and TIM-CLKGR(X2) flies, respectively).

### CLKGR hinders CLK-driven transcriptional oscillations by direct competition with CLK

**Figure 2:**
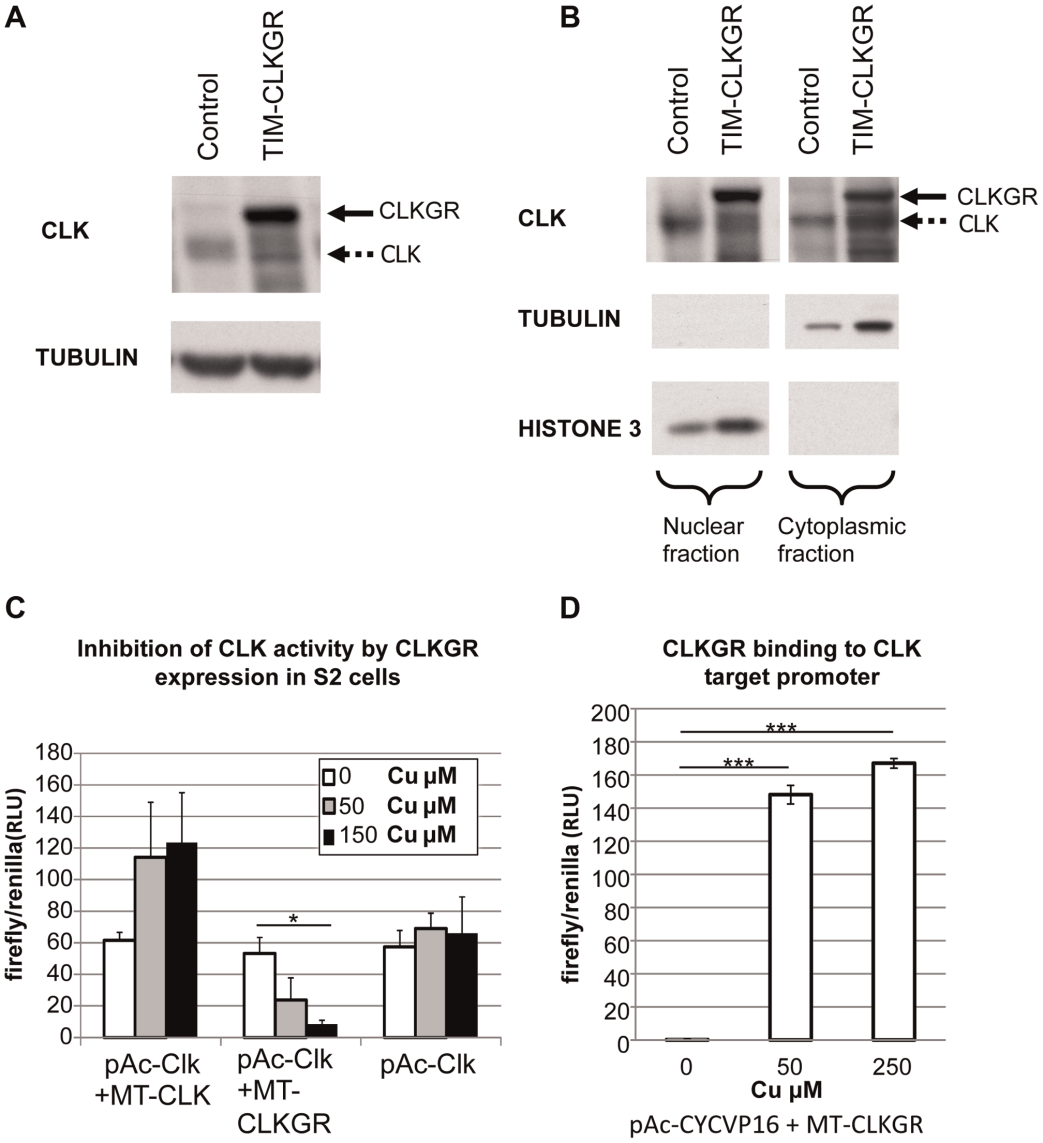
CLKGR is present in the nucleus, and competitively inhibits CLK function. **A.** CLKGR protein is expressed in large amounts in TIM-CLKGR flies. Western blot from fly heads collected at CT15 using an anti-CLK antibody. The assay was performed from TIM-CLKGR flies and control flies (*tim-gal4/+*). Arrows indicate CLK or the CLKGR fusion protein, which can be distinguished by their size. **B.** CLKGR is present in both the nuclei and cytoplasm in TIM-CLKGR flies. Western blot from nuclear and cytoplasmic extracts of control (*tim-gal4/+*) or TIM-CLKGR fly heads collected at CT15. TUBULIN staining is shown as negative control for the nuclear fraction separation and positive control for the cytoplasm fraction. HISTONE-3 staining is shown as positive control for nuclear separation and negative control for the cytoplasm separation. **C.** CLKGR expression can inhibit CLK-mediated activity in *Drosophila* S2 cells. *Drosophila* S2 cells were transfected with *vri-luciferase* reporter plasmid, pAc-CLK plasmid, a plasmid that express CLK or CLKGR under regulation of a copper inducible promoter (metallothionein; MT-CLK or MT-CLKGR respectively), and a plasmid used for controlling transfection efficiency (pCopia-Renilla). No copper or two different amounts of copper were utilized as indicated in the graph. Experiment was done at three separate biological repeats. Plot shows average values of biological duplicates of one representative experimental repeat. Error bars represent standard deviation. One-way Anova was performed to determine statistical significance. *p<0.05. **D.** CLKGR can bind to CLK targets promoters. We induced CLKGR expression in S2 cells using the MT-CLKGR plasmid; in parallel to constant expression of CYCVP16 (from the pAc-CYCVP16 expressing plasmid). CYCVP16 and CLKGR together activate CLK-driven transcription suggesting they bind to CLK targets promoters. Experiment was done at three separate biological repeats. Plot shows average values of duplicates of one representing repeat. Error bars indicate standard deviation. CLK-target activity was measured using a *vri*-luciferase reporter and values were normalized to a transfection control (pCopia-Renilla). T-test was performed to determine statistical significance between time points. ***p<0.001.

One possible explanation for the reduced amplitude of mRNA oscillations in TIM-CLKGR flies is that a fraction of the CLKGR protein localizes to the nucleus and competes for DNA binding with the wild-type CLK. To test this hypothesis we determined the subcellular localization of the fusion protein in TIM-CLKGR flies. We performed a nucleus/cytoplasmic fractionation from fly heads and determined the levels of CLK and CLKGR proteins in each fraction using an anti-CLK antibody. As expected, TIM-CLKGR flies expressed an additional CLK immunoreactive protein of higher molecular weight than the wild-type CLK. The levels of this fusion protein were much higher than the endogenous CLK (Figure 2A). We detected high amounts of the CLKGR fusion protein both in the cytoplasm and in the nuclear fraction (Figure 2B). The presence of CLKGR in the nuclear fraction was not due to cytoplasmic contamination, as tubulin, an exclusive cytoplasmic protein, was only present in the cytoplasmic fraction (Figure 2B).

To determine whether CLKGR could inhibit CLK-mediated transcription, we utilized *Drosophila* S2 cells. These cells do not express CLK, but express CYC at high levels [41]. To test CLKGR/CLK competition, we transfected S2 cells with a *vri*-*luciferase* reporter, a plasmid that expresses CLK at constant levels (pAc-CLK), and a third plasmid in which CLK or CLKGR expression can be induced by addition of copper (pMT-CLK or pMT-CLKGR, respectively). Additional CLK production from the MT-CLK plasmid resulted in a further increase in the levels of the reporter gene expression (Figure 2C, left). However, induction of CLKGR resulted in a dose-dependent reduction of the levels of the reporter expression (Figure 2C, center). This demonstrates that CLKGR can compete with CLK and can partially inhibit CLK-mediated transcription. In *Drosophila* S2 cells CLKGR is incapable of activating CLK-mediated transcription [42]. We postulated that the fraction of the CLKGR protein pool that translocate to the nucleus forms CLKGR-CYC dimers that are inactive due to steric inhibition of the CLK transactivation domain by the GR domain. However, a transcriptionally inactive CLK could activate transcription if co-expressed with an artificial CYC protein carrying a VP16 transcriptional activation domain (CYCVP16 fusion [14]). As an indication for CLKGR binding to the DNA, we determined whether the CLKGR-CYCVP16 dimer could activate transcription. As previously described [14], CYCVP16 alone was not sufficient to active the luciferase reporter (Figure 2D, left bar). However, CLKGR strongly promoted expression of the reporter in presence of CYCVP16 (Figure 2D, right and middle bars). This demonstrates that CLKGR can translocate to the nucleus and bind to CLK-target sites although it cannot activate transcription *per se*.

### TIM-CLKGR flies display lower amplitude protein oscillations than wild-type flies

**Figure 3:**
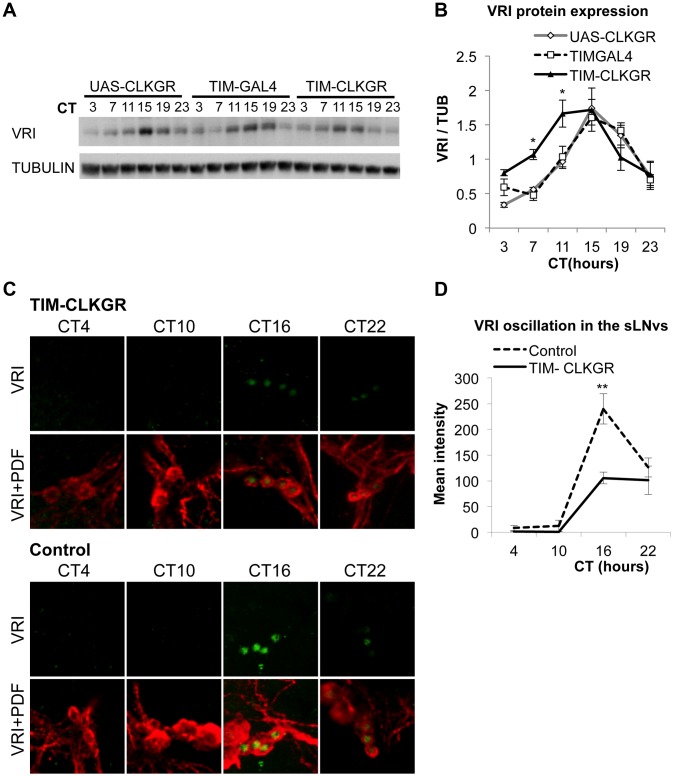
TIM-CLKGR flies display low amplitude circadian protein oscillations. **A.** VRI levels oscillate in TIM-CLKGR flies with lower amplitude. A representative VRI Western blot from TIM-CLKGR and control (*tim-gal4/+* and *UAS-ClkGR/+*) fly heads. We collected the samples at 6 time points during the first day in constant darkness (DD1). **B.** Quantification of VRI oscillations in TIM-CLKGR flies. Western blot staining intensity (Quantification using ImageJ), normalized to tubulin. Average values of three independent biological repeats. Error bars represent SEM. One way ANOVA was performed to determine the statistical significance of the different between VRI levels of TIM-CLKGR and control flies, at CT 7 and CT11 *p<0.05. **C.** Expression of CLKGR decreases the amplitude of VRI oscillation in circadian neurons. VRI (green) and PDF (red) immunostaining of the small Lateral Neurons ventral (sLNvs) from TIM-CLKGR and control (*tim-gal4/+*) fly brains. We collected samples at four time points during the first day in constant darkness. Pictures from one representative experiments are shown. **D.** Quantification of VRI Immunostaining in the sLNvs in constant darkness. Values are average staining intensity of at least 5 brains from each time point. Error bars represent SEM. Wilcoxon test was performed to determine statistical significance. **p<0.01.

To determine the effect of CLKGR expression on the amplitude of circadian protein oscillations, we determined VRI expression by western blot from TIM-CLKGR and control fly heads collected at six different time points during DD1. TIM-CLKGR flies displayed rhythms in VRI expression, although with lower amplitudes than control flies (Figure 3A and 3B). We also recorded protein oscillation *in vivo* by utilizing the XLG PER-luciferase fusion transgene [43]. These flies carry an in frame fusion of the PER protein with luciferase which is driven by *period* promoter regions. Hence luciferase activity correlates with PER protein levels. TIM-CLKGR flies displayed lower amplitude oscillations in PER-Luciferase levels than wild-type controls (Figure S3). Therefore we concluded that defects in circadian transcription in TIM-CLKGR flies resulted also in low amplitude oscillations at the protein level, at least for VRI and the PER-LUC fusion reporter.

In order to determine whether expression of CLKGR also affects the amplitude of protein oscillations in the brain, we determined VRI levels by immunocytochemistry at four different timepoints in the brain of control and TIM-CLKGR flies. We performed the experiment in the first day in constant darkness (DD1). As observed in the western blot assays from whole heads, TIM-CLKGR brains display oscillation in VRI levels in the sLNvs, although of smaller amplitude than control flies (Figure 3C and Figure 3D).

### TIM-CLKGR flies have strong defects in circadian peripheral oscillators

**Figure 4:**
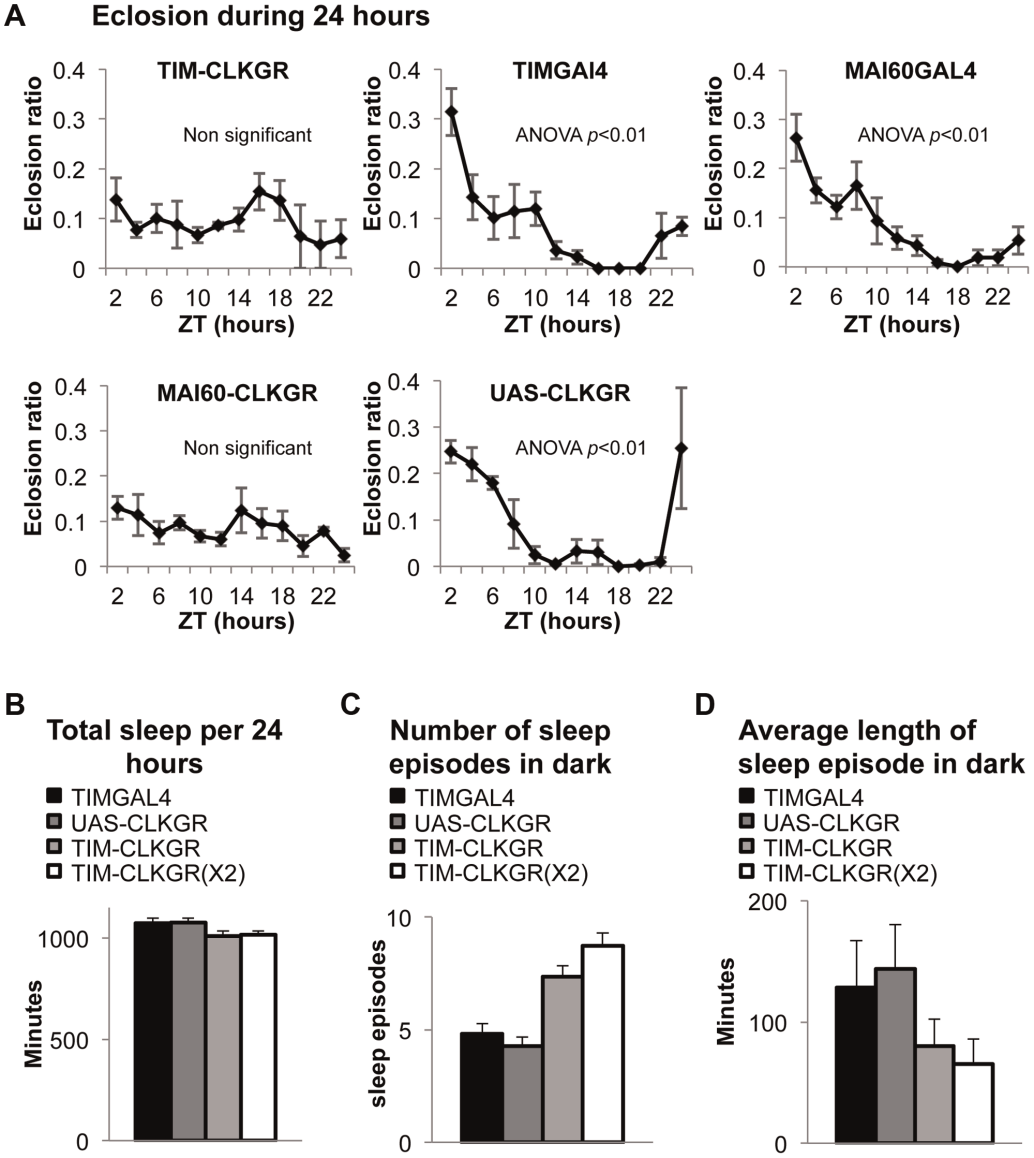
TIM-CLKGR flies display impaired function of peripheral oscillators. **A.** Eclosion circadian gating is absent in TIM-CLKGR flies and in flies that express CLKGR under P{GawB}Mai60 driver (MAI60-CLKGR). The Mai60 driver express predominantly in the prothoracic gland. The eclosion ratio is calculated by determining the portion of flies that emerged in two hours intervals over the total amount of flies that emerged in 24 hours. The experiments were performed in TIM-CLKGR and MAI60-CLKGR flies and for the control lines UAS-CLKGR (UAS-*ClkGR/+*), TIMGAL4 (*tim-gal4/+*) and MAI60GAL4 (flies that carry the P{GawB}Mai60 insertion). Values are the means of 3 or 4 biological repeats (20 to 32 flies in each repeat) Error bars represent SEM. One-way Anova was performed to determine statistical significance of the differences between timepoints, for TIMGAL4, UAS-CLKGR and MAI60GAL4. *p*<0.01. Experiment was performed in LD conditions **B.** Total sleep is not affected in TIM-CLKGR flies. Male flies were kept in 12∶12 LD conditions. Sleep was measured for 5 days. Sleep data was analyzed using pySolo software. Fly strains: TIM-CLKGR (n = 35), TIM-CLKGR(X2) (n = 32) and control flies TIMGAL4 (*tim-gal4/+*,n = 28) and UAS-CLKGR (UAS-*ClkGR/+*,n = 31). Values represent means and errors bars represent SEM. **C.** Expression of CLKGR leads to a dose dependent increase in the number of sleep episodes during the dark phase. Experimental conditions and genotypes as described in B. One-way Anova was performed to determine statistical significance of the differences between fly strains. *p*<0.0001. **D.** Expression of CLKGR leads to a dose dependent decrease in the average length of the sleep episodes during the dark phase. Conditions and genotypes are as described in B. One-way Anova was performed to determine statistical significance of the differences between fly strains, *p*<0.0001.

TIM-CLKGR flies offer an opportunity to determine the importance of transcriptional oscillation amplitude on circadian physiology. In order to test the effect on a peripheral circadian clock, we assayed eclosion timing. A specialized structure, the prothoracic gland, is responsible for the generation of eclosion circadian gating [31], [33]. Wild-type flies have strong eclosion circadian gating either in LD or DD conditions (Figure 4A, and Figure S4A and S4B). However, both LD and DD eclosion rhythms are completely absent in TIM-CLKGR flies (Figure 4A and Figure S4A). In order to determine whether these defects arose from dampening of oscillations in the brain or in the prothoracic gland, we generated flies in which CLKGR expression was restricted to the master pacemaker neurons in the fly brain, the *pdf*-expressing cells, or to the peripheral gland driving eclosion (the prothoracic gland). Whereas expression of CLKGR in the *pdf*-expressing cells in the fly brain did not affect circadian eclosion rhythms (Figure S4B), expression of CLKGR in the prothoracic gland (by the use of the GAL4 driver *Mai60* [33]) was sufficient to suppress daily eclosion rhythms (Figure 4A). Therefore we concluded that the impairment of the eclosion rhythms observed in TIM-CLKGR flies is due to expression of *CLKGR* in the prothoracic gland.

We then determined whether sleep is also affected in TIM-CLKGR flies. Although sleep and circadian behavior are intrinsically linked, sleep is considered a “peripheral-like” output of the circadian clock, despite the fact that it can be controlled by a subgroup of the *pdf*-expressing neurons, the large LNv cells [44]–[47]. We analyzed the sleep behaviors of TIM-CLKGR, TIM-CLKGR(X2), and control flies. As shown in Figure 4B, total sleep was not affected by expression of CLKGR transgenes, but we observed a dramatic effect on the number of sleep episodes during the dark phase, which was dependent in the dose of expressed CLKGR protein (Figure 4C). The significant increase in the number of sleep episodes in TIM-CLKGR flies was accompanied with an equally dramatic decrease in the length of each sleep episode (Figure 4D). From these experiments we concluded that high amplitude rhythms in CLK-driven transcription are necessary for circadian eclosion as well as for normal sleep consolidation.

### TIM-CLKGR flies display quasi-normal locomotor activity rhythms

We next evaluated whether TIM-CLKGR flies have defects in locomotor activity rhythms. Control flies display strong behavioral rhythms both in LD and DD conditions (Table 1). Surprisingly, TIM-CLKGR flies also display rhythmic locomotor activity (Table 1). This indicates that the circadian system driving locomotor rhythms performs well even when the amplitudes of CLK-driven transcriptional oscillations are decreased by approximately 50%, as in TIM-CLKGR flies. On the other hand, most TIM-CLKGR(X2) flies show disrupted circadian rhythms (they are either arrhythmic or rhythmic with very low rhythmic power, see Table 1). Therefore we concluded that the circadian brain oscillator is not able to function when further dampening occurs (e.g. in TIM-CLKGR (X2) flies). It is possible that the opposite results obtained in the eclosion and behavioral assays are due to different thresholds of the assays (i.e. due to the different number of days utilized in the analysis). To rule out this possibility, we utilized a similar timeframe and statistical analysis for determining daily changes in control and TIM-CLKGR flies. As with the previous analysis, we found that while control flies display daily rhythms in both eclosion and locomotor activity, TIM-CLKGR flies have significant differences only in the behavioral assay (Figure S4C, D and E).

**Table 1:** Behavioral characterization of flies expressing CLKGR.

DD1-10
Genotype	Period of Rhythmic	Rhythmic %	Arrhythmic %	Power	N
TIM-CLKGR	24.7(±0.20)	90.3	9.7	525.6(±54.9)	31
TIM-CLKGR(X2)	25.7(±0.48)	45.2	54.8	159.2(±54.9)	31
UAS-CLKGR	23.8(±0.06)	100	0	837.3(±33.9)	31
TIM-GAL4	24.3(±0.08)	96	4	466.2(±41.2)	25

Behavioral analysis of flies maintained for 10 days in constant darkness (DD 1–10). Fly strains: TIM-CLKGR (*tim-gal4*; UAS-*ClkGR*), TIM-CLKGR(X2) (*tim-gal4*; UAS-*ClkGR/*UAS-*ClkGR*), TIMGAL4 (*tim-gal4/+*) and UAS-CLKGR (UAS-*ClkGR/+*). Period of rhythmic flies, rhythmic flies percentage and average power were calculated by chi square power p<0.05. SEM is shown in brackets.

The different effects provoked by the expression of the CLKGR fusion protein in the central and peripheral oscillators is reminiscent of the molecular and behavioral phenotypes of *cryptochrome* (*cry*) mutant flies, which lack the main circadian photoreceptor [48], [49]. These flies display overall low amplitude mRNA oscillations in fly heads, which are due to desynchronization and not to low amplitude oscillations in individual circadian oscillators [50]. Therefore we assessed whether TIM-CLKGR flies have normal circadian photoreception during development and adulthood. Indeed, TIM-CLKGR flies that were raised in LD conditions but transferred to constant darkness before eclosion kept the original phase, demonstrating entrainment capability during development (Figure S5A). Moreover, TIM-CLKGR flies had normal Phase Response Curves (PRC), demonstrating normal CRY function in adult flies (Figure S5B). Therefore the effects observed in TIM-CLKGR flies are not due to inhibition or inactivation of CRY.

### TIM-CLKGR display dampened brain transcriptional oscillations that lead to weaker rhythms after many days in constant conditions

**Figure 5:**
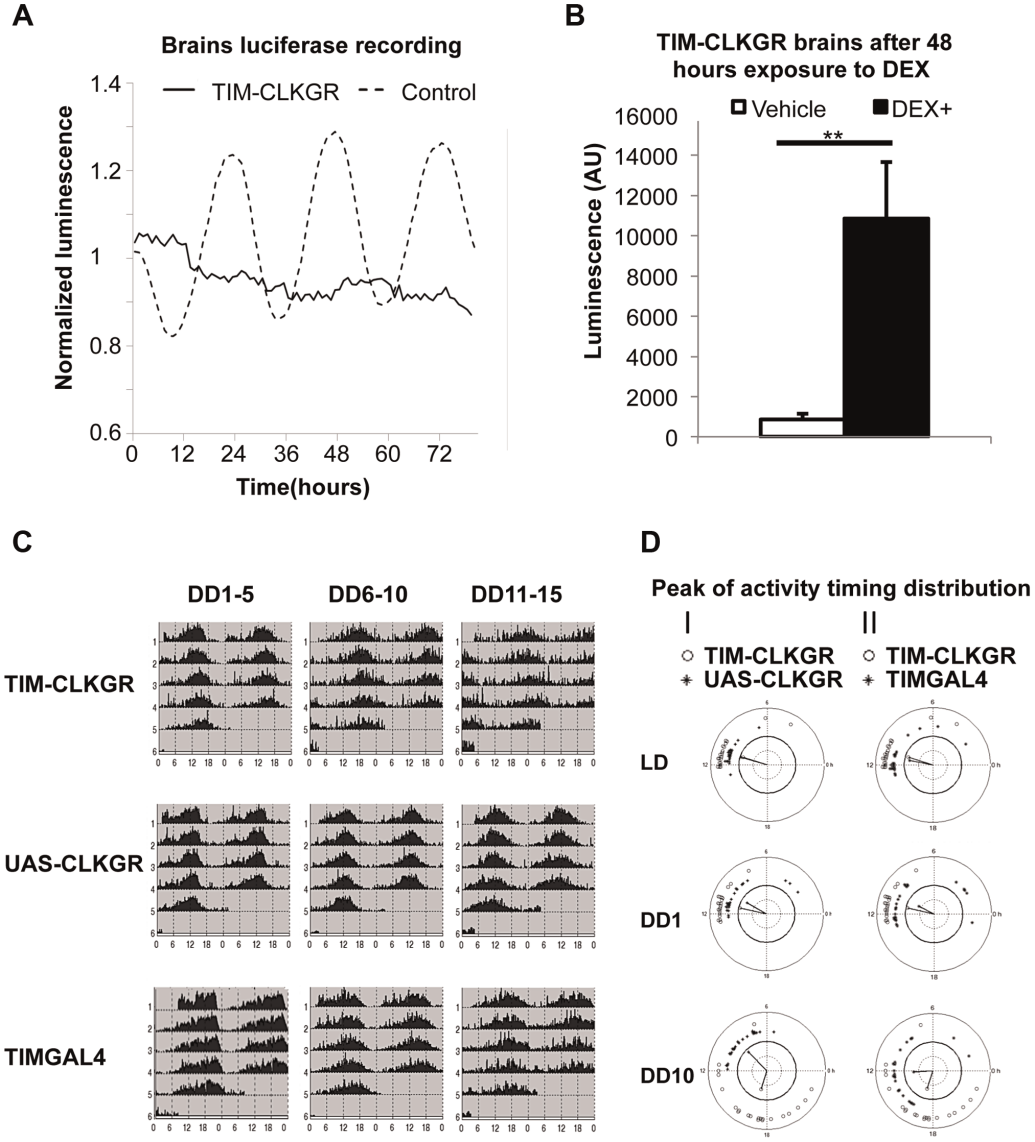
TIM-CLKGR flies display quasi-normal locomotor activity rhythms despite impaired transcriptional oscillations in the brain. **A.** Expression of CLKGR leads to impaired transcriptional oscillations in brains in culture. Luciferase recordings from cultured isolated brains of TIM-CLKGR and control flies. In both cases, we assayed females flies; n = 8 for each strain, brains were maintained at 12 hours light, 12 hours dark light regime (LD). A representative experiment (out of three) is showed. **B.** Addition of dexamethasone induces CLK-driven transcription in cultured TIM-CLKGR brains. Same TIM-ClKGR brains as A. Luminescence mean values after 48 hours exposure to dexamethasone (DEX) (DEX n = 8, vehicle n = 7). A representative experiment (out of three) is showed. Error bars represent SEM. Wilcoxon test was performed to determine statistical significance **p<0.01. **C.** TIM-CLKGR flies display less robust rhythmic behavior after long time in constant darkness. Average locomotor activity of TIM-CLKGR and control flies during five days periods in constant darkness (DD). Behavior was plotted for the first 5 days in constant darkness (DD1–5) days 6 to 10 in constant darkness (DD6–10) and days 11 to 15 in constant darkness (DD11–15). Fly strains: TIM-CLKGR, TIMGAL4 (*tim-gal4/+*) and UAS-CLKGR (UAS-*ClkGR/+*) (rhythms data and flies numbers are shown in Figure S6.) **D.** TIM-CLKGR flies display spread peaks of activity after prolonged times in constant darkness. We plotted the distribution of the activity peaks of individual flies in a circular chart. We did so at different times after starting the experiment: the last day of 12∶12 LD conditions, first day in constant darkness (DD1) and day 10 in constant darkness (DD10). White circles represent TIM-CLKGR individual flies. I) Black dots represent UAS-CLKGR (UAS-*ClkGR/+*) individual flies. II) Black dots represent TIMGAL4 (*tim-gal4/+*) individual flies.

One possible explanation for the resistance of the brain circadian oscillator to the expression of CLKGR is that this fusion protein does not have the same molecular effect in the fly brain as it does in peripheral systems. Although we observed lower amplitude protein oscillations in the brains of TIM-CLKGR flies (Figure 3C, D), we decided to further test this possibility. For doing so, we evaluated transcriptional rhythms in cultured fly brains using a *tim-luciferae* reporter. Control *tim-luciferase* fly brains displayed strong rhythms in LD (Figure 5A, dotted line). TIM-CLKGR brains displayed non-oscillating levels of the transcriptional reporter similar to the patterns displayed by whole TIM-CLKGR flies or cultured fly wings of flies from this genotype (Figure 5A solid line, compare with Figure 1A). In order to ensure that constant low levels of the luciferase reporter in TIM-CLKGR flies are not due to death of the tissue, we added dexamethasone to the TIM-CLKGR brains and followed CLK-driven transcription using the same *tim-luciferase* reporter. TIM-CLKGR cultured fly brains treated with dexamethasone displayed significantly increased levels of CLK-driven transcription relative to untreated brains (Figure 5B). These results are in agreement with the VRI immunocytochemistry profiles (Figure 3C,D) and demonstrate that expression of CLKGR leads to dampened transcriptional oscillations in the brain.

Given these results, we decided to more stringently analyze whether TIM-CLKGR flies displayed any circadian behavioral defects. For doing so, we recorded locomotor activity of control and of TIM-CLKGR flies for more extended times in constant darkness (DD). We computed the percentage of rhythmic flies, the period, and the power, as previously described [51] in three time intervals (DD 1 to 5, 6 to 10, and 11 to 15). Control flies displayed very strong rhythms throughout the experiment with more than 90% of flies displaying rhythmic behavior (Figure 5C and S6). Although most TIM-CLKGR flies were rhythmic even after 15 days in DD, we consistently observed that these flies displayed weaker rhythms and more diverse peak phases than control flies (Figure 5C and 5D). Therefore, we concluded that high amplitude transcriptional oscillations are necessary to maintain robust circadian locomotor activity for long periods in absence of environmental cues.

### The capacity of the brain oscillator to overcome low amplitude transcriptional oscillations depends on synchronous amplitude across the circadian neuronal network and the neuropeptide PDF

**Figure 6:**
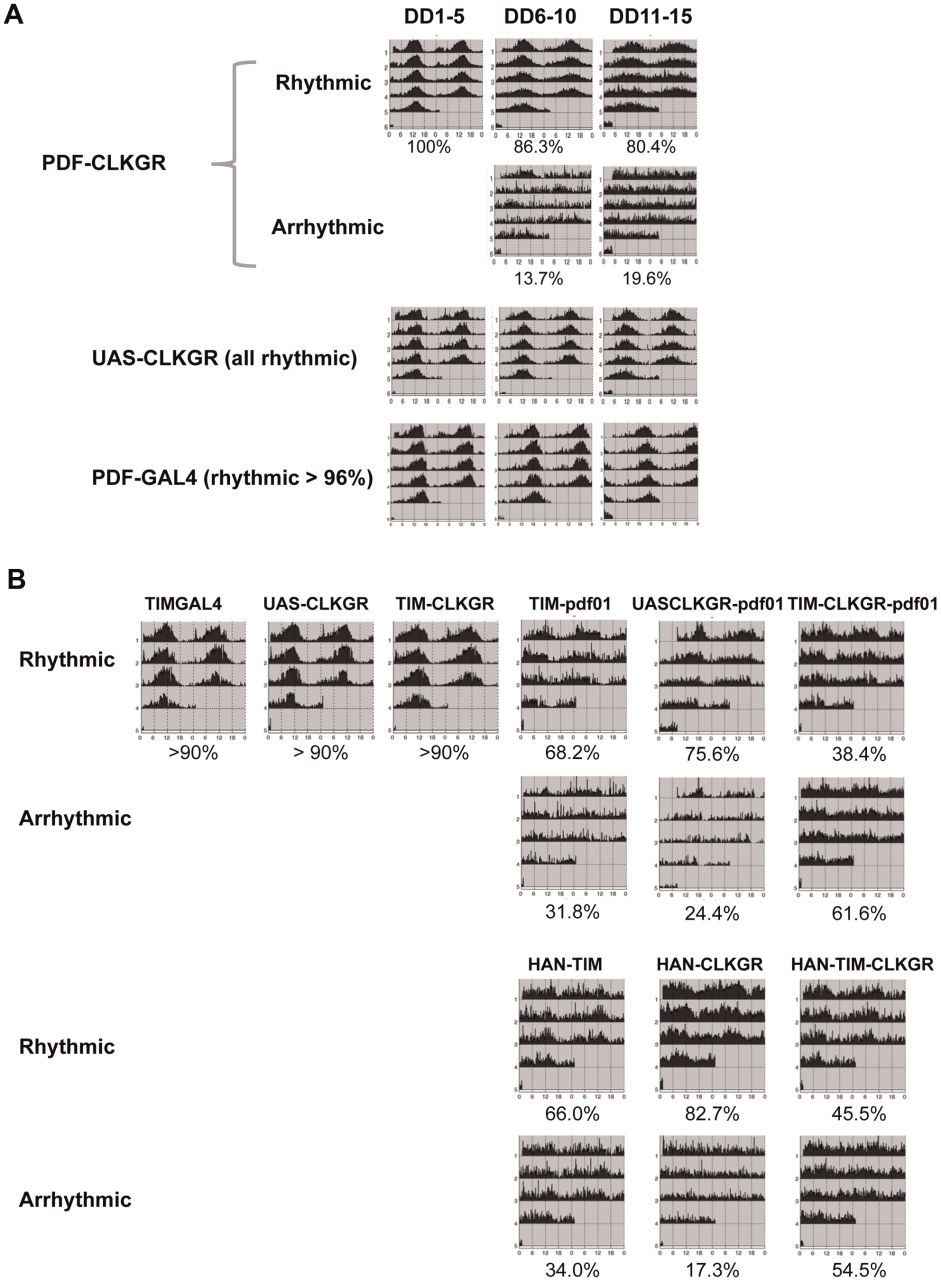
Coordinate amplitude between neuronal groups and the neuropeptide PDF are necessary for compensation of low amplitude molecular oscillations. **A.** Expression of CLKGR in the *pdf*-expressing cells (PDF-CLKGR flies) impairs circadian locomotor behavior. Average of locomotor activity of PDF-CLKGR and control flies during five days periods in constant darkness (DD). Behavior was plotted for the first 5 days in DD (DD1–5), days 6 to 10 (DD6–10) and days 11 to 15 (DD11–15). Fly strains: PDF-CLKGR (*pdf-ga4/+* UAS-*ClkGR/+*), PDFGAL4 (*pdf-ga4/+*) and UAS-CLKGR (UAS-*ClkGR/+*). Rhythms data and flies numbers are shown in Figure S6. Rhythmic percentages were calculated using chi square power. p<0.05. **B.** *Han (Pdf* receptor mutants) and *pdf^01^* mutations genetically interact with expression of the *ClkGR* transgene. Actograms show average locomotor activity at day 2 to day 5 in constant darkness (DD2–DD5). Fly strains: TIM-CLKGR, TIMGAL4 (*tim-gal4/+*), UAS-CLKGR (UAS-*ClkGR/+*), TIM-pdf01 (*tim-gal4/+;pdf^01^*), UASCLKGR-pdf01 (UAS-*ClkGR/+ pdf^01^*), TIM-CLKGR-pdf01 (*tim-gal4/+*;UAS-*ClkGR/+-pdf^01^*), HAN-TIM (*han3369;tim-gal4/+*), HAN-CLKGR (*han3369*;;UAS-*ClkGR/+*), HAN-TIM-CLKGR (*han3369*;*tim-gal4/+*;UAS-*ClkGR/+*). Rhythms data and flies numbers are shown in Figure S9. Rhythmic percentages were calculated by chi square power p<0.05.

The observation that CLKGR expression results in mild behavioral phenotypes, suggest a brain-specific mechanism operating in TIM-CLKGR to buffer the significant decrease in molecular oscillations. In order to investigate this possibility, we determined the behavior of flies in which the CLKGR fusion protein is expressed in different subsets of the circadian neuronal network. First, we generated flies expressing the CLKGR fusion protein only in the *pdf*-expressing cells, (referred to as PDF-CLKGR). Expression of CLKGR in the LNvs alone had strong effects on the percentage of rhythmic flies late in DD (Figure 6A and Figure S6). We then combined the *tim-gal4* driver with cell-specific GAL80-expression transgenes in order to restrict GAL4 activity to different subsets of *tim*-expressing cells (specifically the TIM^+^CRY^−^ and TIM^+^PDF^−^ cells, as previously done by Stoleru et al [24]). Interestingly, expression of GAL80 either in the PDF or CRY-positive neurons improves the mild behavioral phenotype observed in TIM-CLKGR flies in late DD (Figure S7). These results verified the importance of the *pdf*-expressing cells for mediating the robustness of circadian behavior (see below and discussion).

To determine whether the ability of the TIM-CLKGR flies to remain rhythmic is mediated by the neuropeptide PDF, we determined whether flies mutant for components of the PDF-signaling pathway are especially sensitive to expression of the CLKGR transgene. For doing so, we generated TIM-CLKGR flies, which also carry null mutations for the neuropeptide PDF (*pdf^01^*; TIM-CLKGR-pdf^01^ flies) or the PDF Receptor (*Han* mutant flies; HAN-TIM-CLKGR flies). Both *pdf01* and *Han* mutants lose rhythmicity only after several days in DD [18], [21]–[23]. As expected, more than 70% of the *pdf^01^* and the *Han* mutant flies are rhythmic during the first days in DD (Figure 6B and Figure S8). Interestingly, expression of CLKGR in a *pdf* or *Han* mutant backgrounds resulted in a dramatic reduction of the number of rhythmic flies (Figure 6B and Figure S8). Interestingly, the interaction between the CLKGR transgene and the *pdf* signaling pathway is specific, as we did not observed a similar genetic interaction between the CLKGR transgene and the *per^L^* mutation (Figure S9). These results clearly demonstrate that PDF-mediated communication is an essential mechanism mediating the resilience of the brain circadian oscillator to the dampening of circadian transcriptional rhythms provoked by the expression of the CLKGR fusion protein.

## Discussion

In this study, we used the CLKGR fusion protein in *Drosophila* to determine the relative contribution of high amplitude transcriptional oscillations and neuronal communication for robust circadian behavior. Expression of the CLKGR fusion protein in *tim*-expressing cells decreased more than 50% the amplitude of circadian transcriptional oscillations. The impaired transcriptional oscillations lead to low amplitude protein oscillations, which were not sufficient to drive outputs of peripheral oscillators like eclosion rhythms. However, circadian locomotor behavior remained rhythmic. This difference was likely due to intercellular interactions between the circadian neurons in the brain that buffer the low amplitude transcriptional oscillations. Despite this compensation, TIM-CLKGR flies display weaker behavior rhythms after many days in constant darkness. We demonstrated that the compensatory mechanism is dependent on the relationship between the amplitudes of molecular oscillations in different neuronal clusters, especially between the *pdf*-expressing neurons and the rest of the circadian network. Lastly, we showed that the neuropeptide PDF is the key factor contributing to the resilience of the brain oscillator to expression of the CLKGR transgene. Dampening of transcriptional oscillations provoked by CLKGR expression in the context of PDF or PDF receptor mutations resulted in arrhythmicity even very early in constant darkness. In sum, our work revealed the importance of high amplitude transcriptional oscillations in *Drosophila* and how these oscillations contribute to the robustness of the brain circadian oscillator.

Many dominant negatives CLK proteins have been used in the past [15], [52]–[54]. In all these cases, the effects on transcriptional oscillations are dramatic (almost no amplitude remaining), and mutants have strong circadian behavioral phenotypes. In these mutants, the CLK levels critical for development, cell viability, and normal physiology are also severely reduced. For example, *Drosophila Clk^Jrk^* and *Clk^AR^* mutants, present abnormal development of the circadian neurons, precluding the assessment of whether the circadian defects are mainly due to impaired oscillations or developmental defects [53], [55]. Our manipulation offers two advantages to address this issue: First, we can titrate the amplitude of CLK-driven oscillations by utilizing flies with different number of *ClkGR* transgenes, and second, our manipulation does not significantly change the overall levels of CLK-driven transcription (see Figure S2A and S2B). We offer strong evidence of the mechanism by which CLKGR partially inhibits CLK-CYC driven transcription. A fraction of the CLKGR fusion protein leaks into to the nucleus and binds to chromatin, inhibiting the action of the endogenous CLK protein (see Figure 2). The inhibitory action of the CLKGR protein can be explained by steric interference of the ligand-binding domain of the glucocorticoid receptor with the CLK activation domain. We were unable to accurately determine how much CLKGR was chromatin-bound. It may be that most is bound and elicits minimal transcriptional activity or that a small fraction is bound and is transcriptionally inactive (or even inhibitory). We were unable to perform the chromatin immunoprecipitation assays that would have addressed the issue due to the low quality of available antibodies against the GR domain. As expression of CLKGR in *Drosophila* S2 cells did not have any effect on CLK-driven transcription, we favor a competitive binding inhibition scenario. Other than the defective activation, the CLKGR fusion protein seems to respond well to cyclic repression by PER as CLKGR flies still displayed some transcriptional oscillations and similar overall levels of CLK-transcriptional targets as wild-type flies.

Low-amplitude CLK-driven transcription leads to lower amplitude oscillations in all circadian transcription, not just in CLK-direct transcriptional targets (Figure 1E). This demonstrates the centrality of transcriptional control for genome-wide mRNA oscillations. This centrality is highlighted by the strong effect of expression of CLKGR in the physiological output of peripheral oscillators (e.g., eclosion). Although some of the core circadian components like PER and TIM are strongly regulated at the post-translational level, our results suggests that most output genes are regulated at the transcriptional and post-transcriptional levels (rather than post-transnationally). Post-transcriptional regulation also cannot be ruled out, as CLK-driven transcription could affect indirectly the oscillation amplitudes of hundreds of genes by regulation of non-coding RNAs or RNA binding proteins.

In addition to promoting high-amplitude protein oscillations, CLK-driven transcription can serve other functions. For example, for genes with long-lived mRNA and/or protein products, direct CLK control ensures that these genes are expressed in circadian tissues. Moreover, in cases of mRNA and/or proteins with very high turnover rates, CLK-dependent control means that functional levels are reached at least once a day. We speculate that this may be the case for sleep control by the circadian system; CLK may directly or indirectly modulate the levels of dopamine-related arousal signals in the brain (e.g. in the large LNvs), which have been shown to be regulated/influenced by the circadian system: [44], [46], [56].

It has been previously postulated that transcriptional rhythms may not be necessary for accurate circadian timekeeping. Our study definitively demonstrates the necessity for high-amplitude transcriptional oscillations for normal circadian output, especially in peripheral tissues. Although some aspects of circadian behavior can be rescued when TIM and PER are expressed at constant levels [57], *per* mRNA oscillations in *Drosophila* and feedback repression in mammals are key for proper circadian control [14], [15]. However, to the best of our knowledge this is the first time transcriptional oscillations have been partially damped in a living organism and their role assessed comprehensively.

Transcriptional oscillations seem to be less important for the brain circadian oscillator. We postulate that in the brain, communication between the circadian neuronal groups can compensate for the dampened transcriptional oscillations. This is not surprising and results obtained in mammals are among the same lines [58]. Mutations in core clock components, which have deleterious effects on transcriptional oscillations in isolated suprachiasmatic nucleus neurons and in peripheral clocks, have mild or no effects on daily locomotor activity patters [38], [59]. This resilience and the general robustness properties of circadian oscillators in the suprachiasmatic nucleus are due to neuron-to-neuron communication [60], [61]. However, in mammals the molecular machinery that drives circadian rhythms in the central and in the peripheral oscillators differs [62], [63], while this does not seem to be the case in flies.

The use of the CLKGR system allowed us to determine until which point the circadian clock can compensate for dampened transcriptional oscillations. For example, the brain oscillator can still function fairly well after reducing the amplitude of oscillations more than 50% (TIM-CLKGR flies) but not after further flattening.

Interestingly, we found that PDF-CLKGR flies display a stronger behavioral phenotype than TIM-CLKGR flies (Figure 6A). We don't think this is due to different levels of expression of the CLKGR proteins in the sLNVs, as *pdf-gal4* and *tim-gal4* drivers express with similar strength in those cells [25]. Moreover, previous results utilizing this driver in combination with UAS-transgenes that affect circadian period like *sgg* or *CYCVP16* also do not support this possibility [14], [64]. Our interpretation is that the compensatory mechanism operating in TIM-CLKGR flies requires that the molecular oscillations in the *pdf*-expressing cells be of equal or higher magnitude than in the rest of the circadian neuronal network. We speculate that this is due to the hierarchical nature of the circadian neuronal network (with the sLNvs being at the top of this hierarchy). Hence, in TIM-CLKGR flies, sLNvs can still set the pace of the circuit and the circadian clock in a *pdf*-pathway dependent way. In PDF-CLKGR flies, low amplitude oscillations in the sLNvs are not enough to drive the rest of the network, likely due to more resistance from the other neuronal groups, which have higher amplitude molecular oscillations than the *pdf*-expressing cells. This is further supported by the fact that we did not observe any behavioral defect when we expressed the CLKGR fusion only in the CRY^+^PDF^−^ or the TIM^+^CRY^−^ cells. The centrality of the sLNvs for the compensatory mechanism is highlighted by the fact that removing PDF signaling eliminates the capacity of TIM-CLKGR flies to keep rhythmic behavioral patterns (Figure 6B and Figure S8).

In sum, our study revealed important differences between the central and peripheral circadian oscillators regarding the dependence on transcriptional oscillations. By dissecting the mechanism mediating the resilience of the brain oscillators, we were able to dissect the contributions of molecular and neuronal network pathways on the generation of robust and coherent behavioral circadian rhythms.

## Materials and Methods

### Fly strains

*Tim-gal4*, *pdf*-*gal4*, *tim-luc*, UAS-*ClkGR*, *per.XLG-luc*, *pdf-gal80*, *cry-gal80 and Per^L^* were previously described [14], [17], [43], [53], [65]–[68] Han3369 and P{GawB}Mai60 [23], [33] lines were obtained from Bloomington stock center.

### Microarrays

Total RNA was extracted using Trizol reagent (Invitrogen). Probe preparation and hybridization, staining and washing of the Affymetrix high-density arrays were carried out as described in the Expression Analysis Technical Manual (Affymetrix).

### Locomotor behavior

We used male flies at all behavior experiment except for the experiment described at figure S8 in which females were used. Flies were monitored using Trikinetics *Drosophila* Activity Monitors (Waltham, MA, USA). Rhythmic flies were determent by chi square power, using Faas software (http://www.inaf.cnrs-gif.fr/ned/equipe03_eng/faasx.html) [51].

### Sleep measurements

Sleep measurements were performed using Trikinetics *Drosophila* Activity Monitors (Waltham, MA, USA). In all the cases we recorded the activity of male flies during 5 days in 12 hours light 12 hours dark light regime (LD) on 1 minute intervals. For analyzing the data we utilized the software pySolo [69].

### Eclosion assay

For assaying eclosion ratio at 12 hours light 12 hours dark regime, we placed individual pupas into behavior tubes. New emerged flies were detected by monitoring movements using Trikinetics *Drosophila* Activity Monitors (Waltham, MA, USA). In order to find eclosion ratios in the first day in constant darkness (DD1), fly populations were entrained in bottles to light regimes of 12 hours light and 12 hours dark for 3 days and then transferred into constant darkness. Adult flies were removed from the bottles at the end of the last light cycle (ZT24) and newly emerged flies were then removed from the bottles and counted every 2 hours.

### Fly brain immunocytochemistry

Flies were entrained for at least 3 days in 12∶12 LD, and then transferred to constant darkness conditions. During DD1 four time points were collected. Whole flies were placed into fixative solution (PBS 4% paraformaldehyde 0.1% triton-x) for 30 minutes in 4°C followed by 2 hours rotation at room temperature. Then flies were transferred to PBS and the brains were dissected, wash 3 times (PBS 0.1% triton-x) and transferred to 30 minutes blocking solution, (PBS 0.1% triton-x 2% horse serum). After 3 more washes, brains were incubated with primary antibody solution overnight, PBS 0.1% triton-x 2% horse serum 1∶3000 G.P anti VRI (gift from Paul Hardin) 1∶1000 Ms anti PDF (gift from Justin Blau). Brains were washed 3 more times and incubated with secondary antibodies solution, PBS 0.1% triton-x 2% horse serum 1∶500 Alexa Fluor 488 Goat anti G.P (invitrogene) 1∶500 Dylight 550 Dnk anti Ms (Abcam), for 1 hour in room temperature. Brains were washed 3 times and mounted in VECTASHIELD mounting medium (VECTOR) on microscope slides. Photos were taken using Eclipse Ti - Nikon confocal microscope in magnitude of ×200. Quantifications were done utilizing NIS-Elements Ar Microscope Imaging Software.

### Real-time monitoring of luciferase activity

Adult male flies and dissected heads wings and brains were cultured in 12∶12 LD conditions, and luciferase was measured as described previously [70].

### Plasmids

Plasmids were described previously: pAc-CYCVP16 [14], MT-CLKGR [71]. vri-luc [72], pAc-clk, Copia Renilla luciferase and tim-luc, [42]. MT-CLK was generated by amplifying the *Clk* ORF by PCR and ligating it into pMT-V5 (Invitrogen) using the enzymes KpnI and NotI.

### S2 cells transfection

*Drosophila melanogaster* Schneider-2 cells were grown at 25°C in Schneider's Medium with L-Glutamine (Biological Industries, Jerusalem, Israel/Invitrogen, Carlsbad, CA, USA) supplemented with 10% fetal bovine serum (GIBCO) and 1% Antibiotic-Antimycotic, GIBCO. Cells were seeded in a 6 well plate. Transfection was performed at 70–90% confluence according to company recommendations (6 µl of TransIT-2020 Transfection reagent, Mirus and 2 µg of total DNA). In all experiments 75 ng of pCopia-Renilla Luciferase plus 50 ng of the Luciferase firefly reporter were used. For the plasmids MT-CLK, MT-CLKGR and pAc-CYCVP16 100 ng were used.

### Luciferase activity assay

Twenty-four hours after transfection, cells were treated with CuSO4 in the indicated doses and after 24 hs of induction, cells were lysed and assayed using the Dual Luciferase Assay Kit (Promega) following the manufacturer's instructions. In the transfections with pAc-CYCVP16 cells were collected 48 hs after transfection.

### Analysis of gene expression by real-time PCR

Total RNA was prepared from adult fly heads (30 heads per sample) using Trizol reagent (Sigma) according to the manufacturer's protocol. cDNA derived from this RNA (using iScript Bio-Rad) was utilized as a template for quantitative real-time PCR performed with the C1000 Thermal Cycler Bio-Rad. The PCR mixture contained Taq polymerase (SYBR green Bio- Rad). tim: 5′-CCTTTTCGTACACAGATGCC-3′, 5′ –GGTCCGTCTGGTGATCCCAG-3′ and 5′-GCTGGCCGATTACAGGATAAC-3′, 5′AGTAAAACAGCGGCACACTCA-3′; vri: 5′- GTCTAATTCTCGCTCCCTCT -3′, 5′- GAACTTTCTTTGTTCGTTGG -3′; Rp49: 5′-TACAGGCCCAAGATCGTGAA-3′, 5′-CCATTTGTGCGACAGCTTAG -3′; and RpS18: 5′CCTTCTGCCTGTTGAGGA- -3′ 5′-TGCACCGAGGAGGAGGTC -3′. Cycling parameters were 95°C for 3 min, followed by 40 cycles of 95°C for 10 s, 55°C for 10 s, and 72°C for 30 s. Fluorescence intensities were plotted versus the number of cycles by using an algorithm provided by the manufacturer. mRNA levels were quantified using a calibration curve based upon dilution of concentrated cDNA. mRNA values from heads were normalized to that from ribosomal proteins 49 (Rp49) and RpS18.

### Western blotting

Fly heads (20 heads per sample) were collected and homogenized in RIPA lysis buffer (50 mM Tris-HCl at pH 7.4, 150 mM NaCl, 1 mM EDTA, 1% NP-40 0.5% Sodium deoxycholate, and 0.1% sodium dodecyl sulfate (SDS), 1 mM DTT, with protease inhibitor cocktail and phosphatase inhibitors). Head lysates were then centrifuged for 10 minutes and the supernatant was boiled with protein sample buffer (Bio-Rad). Samples were resolved by Criterion XT Bis-Tris gels (Bio-Rad). Antibodies used for Western blotting were as follows: anti-CLK (a kind gift from Paul Hardin), anti-VRI (a kind gift from Paul Hardin), anti Tub (DM1A, SIGMA), anti His3 (Abcam). Quantifications were done utilizing Image J software.

### Nuclear/cytoplasmic fractionation

Fly heads were homogenized in a Dounce homogenizer, in the following buffer: 10 mM Hepes pH 7.5, 10 mM KCl, 0.8 M Sucrose, 1 mM EDTA, 0.5 mM DTT, supplemented by protease-inhibitor cocktail (mini complete, Roche) and phosphatase inhibitors. After homogenization, the homogenate was filtered through a column polymer bed support (Bio-Rad unfilled Bio-spin Column 4 minutes 1000 g 4°C) to remove the cuticle. The filtrate was then centrifuged (600 g, 10 minutes 4°C) and the pelleted cell extract were then subjected to nuclear cytoplasmic fractionation. To prepare the cytoplasmic fraction, the cell pellets were re-suspended in cytoplasmic buffer (10 mM Tris HCl pH 8.0, 10 mM KCl, supplemented by protease inhibitor cocktail (mini complete, Roche) and phosphatase inhibitors). Cells were allowed to swell for 15 minutes, and then NP-40 was added to 0.4%, followed by centrifugation (3500 g, 3 minutes 4°C). The supernatant contained the soluble cytoplasmic fraction. The pellets were washed once more with the cytoplasmic buffer before proceeding to nuclear fractionation. For the preparation of the nuclear fraction, the remaining cell pellet was re-suspended in high-salt buffer (50 mM Tris pH 8.0, 5 mM EDTA, 400 mM NaCl, 1% NP-40, 1% Sodium deoxycholate, and 0.025% sodium dodecyl sulfate (SDS), 1 mM DTT supplemented by protease inhibitor cocktail (mini complete, Roche) and phosphatase inhibitors). The nuclear pellet was hard vortex for 30 min at 4°C, and was than centrifuged (1 minute 4°C max speed). The supernatant, which contains the nuclear fraction, was saved. Both fractions were re-suspended in protein sample buffer, heated 5 minutes 95°C.

### Phase Response Curve (PRC)

Flies were entrained to a 12∶12 LD cycle for 4 days. During the fifth dark phase of the cycle, flies groups contains 32 flies were given a 10-min saturating white light pulse (1000 lux) at 13, 15, 17, 19, 21, and 23 h after the last light-on event. A separate control group of 32 flies was not given a light pulse. Flies were then put into DD. The average phase of the locomotor activity peaks after the light pulse was determined and compared with the no-light-pulse control.

### Pre-adult entrainment testing

In order to test pre adult entrainment flies were grown at 12 hours light: 12 hours dark (LD) light regime till pupa stage. Pupas have been placed into behavior tubes in constant darkness (DD). After eclosion, the locomotor activity of the flies was monitored in DD in order determinate the phase of circadian activity.

## Supporting Information

Dataset S1 Microarray expression data from control and TIM-CLKGR flies. RNA extracted from fly heads of Control (UAS-*ClkGR*/+) and TIM-CLKGR flies collected at ZT3 and ZT15 was used to perform Oligonucleotide microarrays as indicated in the method sections. The file contains the Gene name and description (Column A) and the normalized data (columns B–K). For each genotype the amplitude between timepoints was calculated for each probe by computing the ratio between the signal at ZT3 and at ZT15. For each gene we computed the relationship between the amplitudes observed in control and TIM-CLKGR flies (column N). Statistical tests were performed to determine differences of gene expression between timepoints in TIM-CLKGR flies (column O) or control flies (column P).
(XLSX)

Figure S1 CLKGR increases CLK driven transcription when exposed to dexamethasone (DEX). DEX increases CLK driven transcription in TIM-CLKGR isolated fly wings. 0.5 µM DEX exposure increases *tim*-luciferase reporter activity in isolated TIM-CLKGR wings (mean luminescence at 60 hours, average of 16 pair of wing samples for each treatment). Error bars represent SEM. T-test was performed to determine statistical significance. ***p<0.001.
(TIF)

Figure S2 TIM-CLKGR flies display overall similar levels of CLK-target mRNAs despite diminished mRNA oscillations. **A.** Average *vri* expression in control and TIM-CLKGR flies. Values represent the average expression of *vri* from six time points of the experiment describe on Figure 1C. Error bars represent SEM. **B.** Average expression of CLK target genes. Values represent the average expression from two time points of different CLK target genes from the microarray described at Figure 1E and in Dataset S1. Error bars represent SEM.
(TIF)

Figure S3 PER-LUC fusion protein oscillates with lower amplitude in TIM-CLKGR fly wings. Average luciferase readings from fly wings that carry the PER-luc (XLG) transgene. The experiment was performed in Light∶dark (LD) 12∶12 cycles. The genotypes of the strains are: TIM-CLKGR (*XLG*;*tim*-*gal4*/+;UAS-*ClkGR*/+) and Control (*XLG*;*tim-gal4/+*). Values show the average record from 30 pairs of wings. Error bars represent SEM.
(TIF)

Figure S4 CLKGR expression impairs circadian eclosion without much effect in locomotor activity rhythms. **A.** Eclosion circadian gating is impaired in TIM-CLKGR flies in constant darkness conditions. We plotted the ratio between the number of the flies that emerged in two hours intervals and the total amount of flies that emerged in 24 hours for TIM-CLKGR flies and UAS-CLKGR (UAS-*ClkGR/+*) flies. Values are the means of 2 biological repeats (50 to 100 flies in each repeat). Error bars represent SEM. One-way Anova was performed to determine statistical significance of the differences between timepoints, for UAS-CLKGR eclosion. *p*<0.01. Experiment was performed during the first day in constant darkness conditions (DD1). **B.** Expression of CLKGR in the LNvs (PDF expressing cells) does not affect circadian eclosion rhythms. Conditions as in Figure 4A. Flies lines: PDF-CLKGR (*pdf-gal4/+*; UAS-*ClkGR/+*), PDFGAL4 (*pdf-gal4/+*), UAS-CLKGR (UAS-*ClkGR/+*). One way Anova was performed to determine statistical significance of the differences between timepoints. *p*<0.01. **C.** TIM-CLKGR and MAI60-CLKGR flies have strong locomotor activity rhythms and impaired eclosion rhythms. We measured locomotor activity during 24 hours in 12∶12 LD condition, and plot the ratio between the level of locomotor activity in 2 hours intervals and the total levels of locomotor activity in 24 hours. Values for locomotor activity are mean of 3 repeats (flies number in each repeat 27–32). Eclosion ratios are plotted as in Figure 4A. Error bars represent SEM. One-way Anova was performed to determine statistical significance of the differences between timepoints. *p*<0.0001 for locomotor activity and not significant for eclosion profiles. **D. and E.** Control flies UAS-CLKGR (UAS-*ClkGR/+*), TIMGAL4 (*tim-gal4/+*) and MAI60GAL4 (flies that carry the P{GawB}Mai60 insertion) show circadian eclosion and circadian locomotor activity ratios. Conditions as in C. One-way Anova was performed to determine statistical significance of the differences between timepoints. *p*<0.01 for eclosion and *p*<0.0001 for locomotor activity.
(TIF)

Figure S5 TIM-CLKGR flies have normal photoreception. **A.** TIM-CLKGR flies can be synchronized in the pre-adult stage. We entrained TIM-CLKGR and control flies during the larval and pupal stages to LD cycles and transferred to behavioral tubes directly in constant darkness (DD). We then assayed locomotor activity rhythms. We observed that these rhythms are synchronized with the larval/pupal entrainment light regime. Right plot flies average locomotor activity during ten days period after eclosion, left plot average activity per day. TIM-CLKGR and control (*tim-gal4/+*) flies were assayed. **B.** Phase Respond Curve (PRC) is indistinguishable between control and TIM-CLKGR flies. TIM-CLKGR phase respond curve is similar to control (*tim-gal4/+*). The time onset of the photic stimuli was plotted on the X-axis (ZT, in hours). The phase response was plotted on the Y-axis as the difference (in hours) from the phase of untreated flies. Mean of two repeats. Error bars represents standard deviation.
(TIF)

Figure S6 Behavioral characterization of CLKGR flies and control flies during 15 day in DD. Table sections show data for: DD 1–5, DD 6–10 and DD 11–15. Average period of rhythmic flies, rhythmic flies percentage and average power were calculated by chi square power. p<0.05. SEM is shown in brackets. Fly strains: TIM-CLKGR, PDF-CLKGR (*pdf-gal4/+*;UAS-*ClkGR/+*), UAS-CLKGR (UAS-*ClkGR/+*), TIMGAL4 (*tim-gal4/+*), PDFGAL4 (*pdf-gal4/+*), TIM-CLKGR-CRYGAL80 (*tim-gal4/+*;UAS-*ClkGR,cry-gal80/+*), TIM-CLKGR-PDFGAL80 (*tim-gal4/+*;UAS-*ClkGR,pdf-gal80/+*), TIM-CRY-GAL80 (*tim-gal4/+;cry-gal80/+*) and PDF-GAL80 (*pdf-gal80*).
(TIF)

Figure S7 Expression of CLKGR in the TIM^+^CRY^−^ or TIM^+^PDF^−^ cells does not result in the behavioral defects observed in TIM-CLKGR flies. **A.** TIM-CLKGR flies display less robust rhythmic behavior after long time in constant darkness than TIM-CRY-GAL80 (*tim-gal4/+*;UAS-*ClkGR,cry-gal80/+*) and TIM-CLKGR-PDFGAL80 (*tim-gal4*;UAS-*ClkGR,pdf-gal80/+*). Behavior was plot for the first 5 days in constant darkness (DD1–5), days 6 to 10 in constant darkness (DD6–10) and days 11 to 15 in constant darkness (DD11–15) (rhythms data and flies numbers are shown in Figure S6). **B.** TIM-CLKGR flies display more spread peaks of activity after prolonged times in constant darkness than TIM-CRY-GAL80 and TIM-CLKGR-PDFGAL80 flies. Peak of activity of each fly are plotted in circular chart for the last day of 12∶12 LD conditions before transferred to constant darkness, first day in constant darkness (DD1) and day 10 in constant darkness (DD10). White circles represent TIM-CLKGR individual flies. I) Black dots represent TIM-CRY-GAL80 (*tim-gal4/+*;UAS-*ClkGR,cry-gal80/+*) individual flies. II) Black dots represent TIM-CLKGR-PDFGAL80 (*tim-gal4*;UAS-*ClkGR,pdf-gal80/+*) individual flies.
(TIF)

Figure S8 Behavioral characterization of TIM-CLKGR flies with *pdf* or *pdfr* (*Han*) null mutation. Rhythmicity results from day 2 to day 5 in constant darkness (DD2 to DD5). Fly strains: TIM-CLKGR, TIMGAL4 (*tim-gal4/+*), UAS-CLKGR (UAS-*ClkGR/+*), TIM-pdf01 (*tim-gal4/+;pdf^01^*), UASCLKGR-pdf01 (UAS-*ClkGR/+pdf^01^*), TIM-CLKGR-pdf01 (*tim-gal4/+*;UAS-*ClkGR/+pdf^01^*), HAN-TIM (*han3369;;tim-gal4/+*), HAN-CLKGR (*han3369*;;UAS-*ClkGR/+*), HAN-TIM-CLKGR (*han3369*;*tim-gal4/+*;UAS-*ClkGR/+*). Average period of rhythmic flies, rhythmic flies percentage and average power were calculated by chi square power p<0.05. SEM is shown in brackets.
(TIF)

Figure S9 Expression of CLKGR does not genetically interact with the *perL* mutation. Results of locomotor activity of female flies at 10 days in constant darkness (DD1–10). TIMGAL4 (*tim-gal4/+*), UAS-CLKGR (UAS-*ClkGR/+*), TIM-CLKGR, PERL-TIMGAL4 (*per^L^/+*;*tim-gal4/+*), PERL-UAS-CLKGR(*per^L^/+*;;UAS-*ClkGR/+*), PERL-TIM-CLKGR (*per^L^/+;tim-gal4/+*;UAS-*ClkGR/+*). Average period of rhythmic flies, rhythmic flies percentage and average power were calculated by chi square power p<0.05. SEM is shown in brackets.
(TIF)
