# Supplementary figures and images for: Synergistic Interactions between the Molecular and Neuronal Circadian Networks Drive Robust Behavioral Circadian Rhythms in Drosophila melanogaster
Source: PLoS Genet. 2014 Apr 3;10(4):e1004252. doi: 10.1371/journal.pgen.1004252 (PMC3974645; doi:10.1371/journal.pgen.1004252)

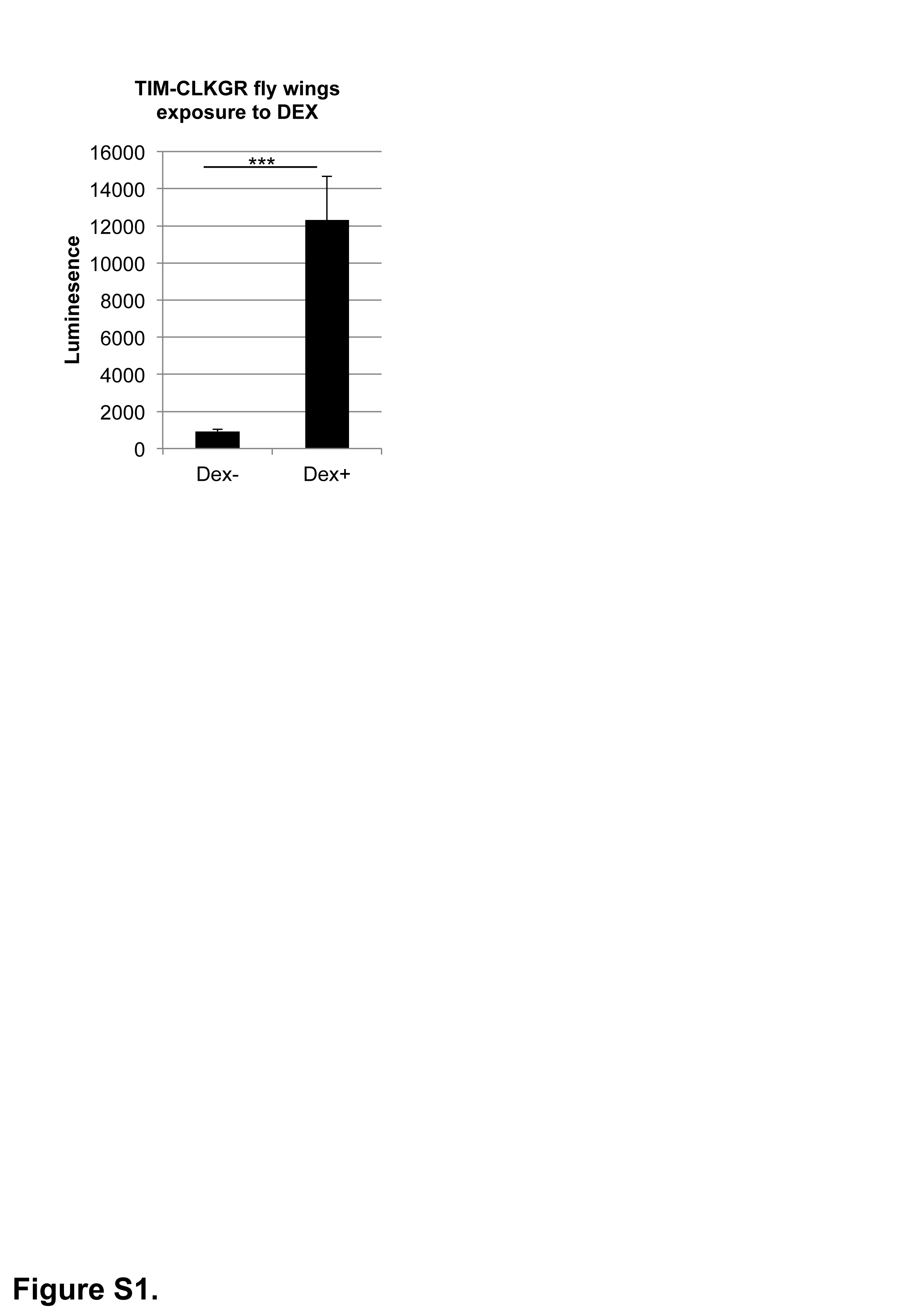

Supplement: Figure S1 — CLKGR increases CLK driven transcription when exposed to dexamethasone (DEX). DEX increases CLK driven transcription in TIM-CLKGR isolated fly wings. 0.5 µM DEX exposure increases tim-luciferase reporter activity in isolated TIM-CLKGR wings (mean luminescence at 60 hours, average of 16 pair of wing samples for each treatment). Error bars represent SEM. T-test was performed to determine statistical significance. ***p<0.001. (TIF) [file pgen.1004252.s002.tif]

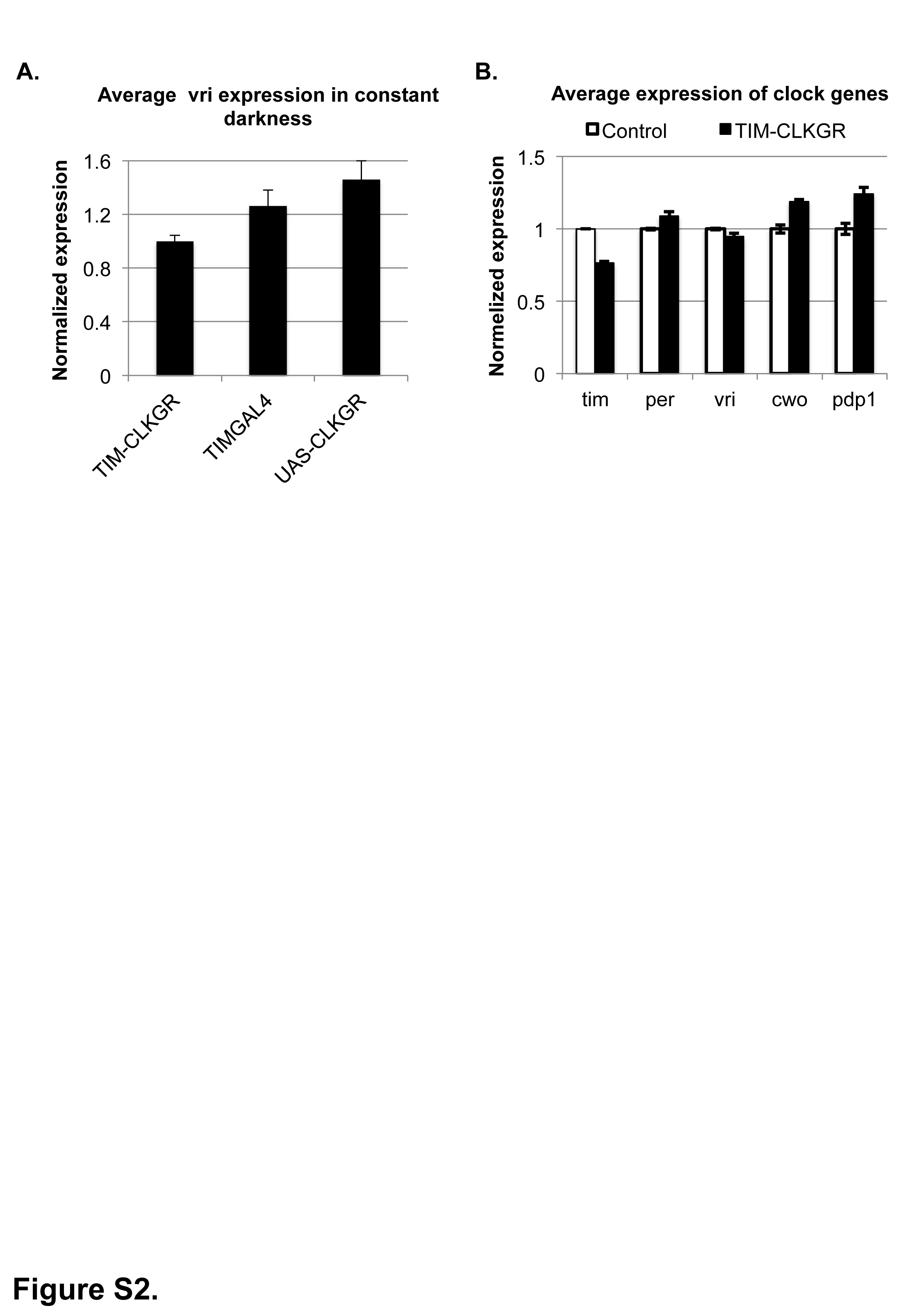

Supplement: Figure S2 — TIM-CLKGR flies display overall similar levels of CLK-target mRNAs despite diminished mRNA oscillations. A. Average vri expression in control and TIM-CLKGR flies. Values represent the average expression of vri from six time points of the experiment describe on Figure 1C. Error bars represent SEM. B. Average expression of CLK target genes. Values represent the average expression from two time points of different CLK target genes from the microarray described at Figure 1E and in Dataset S1. Error bars represent SEM. (TIF) [file pgen.1004252.s003.tif]

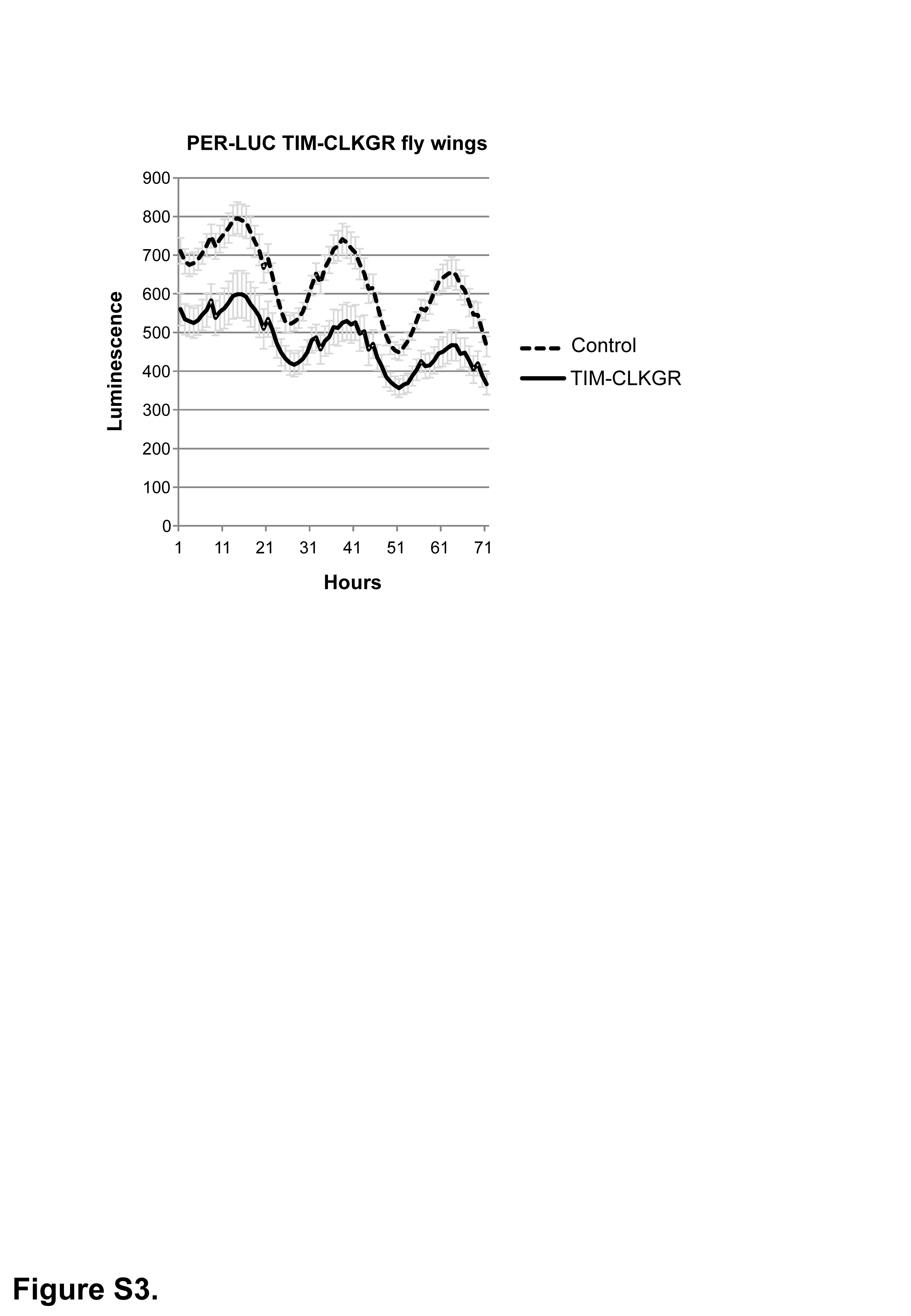

Supplement: Figure S3 — PER-LUC fusion protein oscillates with lower amplitude in TIM-CLKGR fly wings. Average luciferase readings from fly wings that carry the PER-luc (XLG) transgene. The experiment was performed in Light∶dark (LD) 12∶12 cycles. The genotypes of the strains are: TIM-CLKGR (XLG;tim-gal4/+;UAS-ClkGR/+) and Control (XLG;tim-gal4/+). Values show the average record from 30 pairs of wings. Error bars represent SEM. (TIF) [file pgen.1004252.s004.tif]

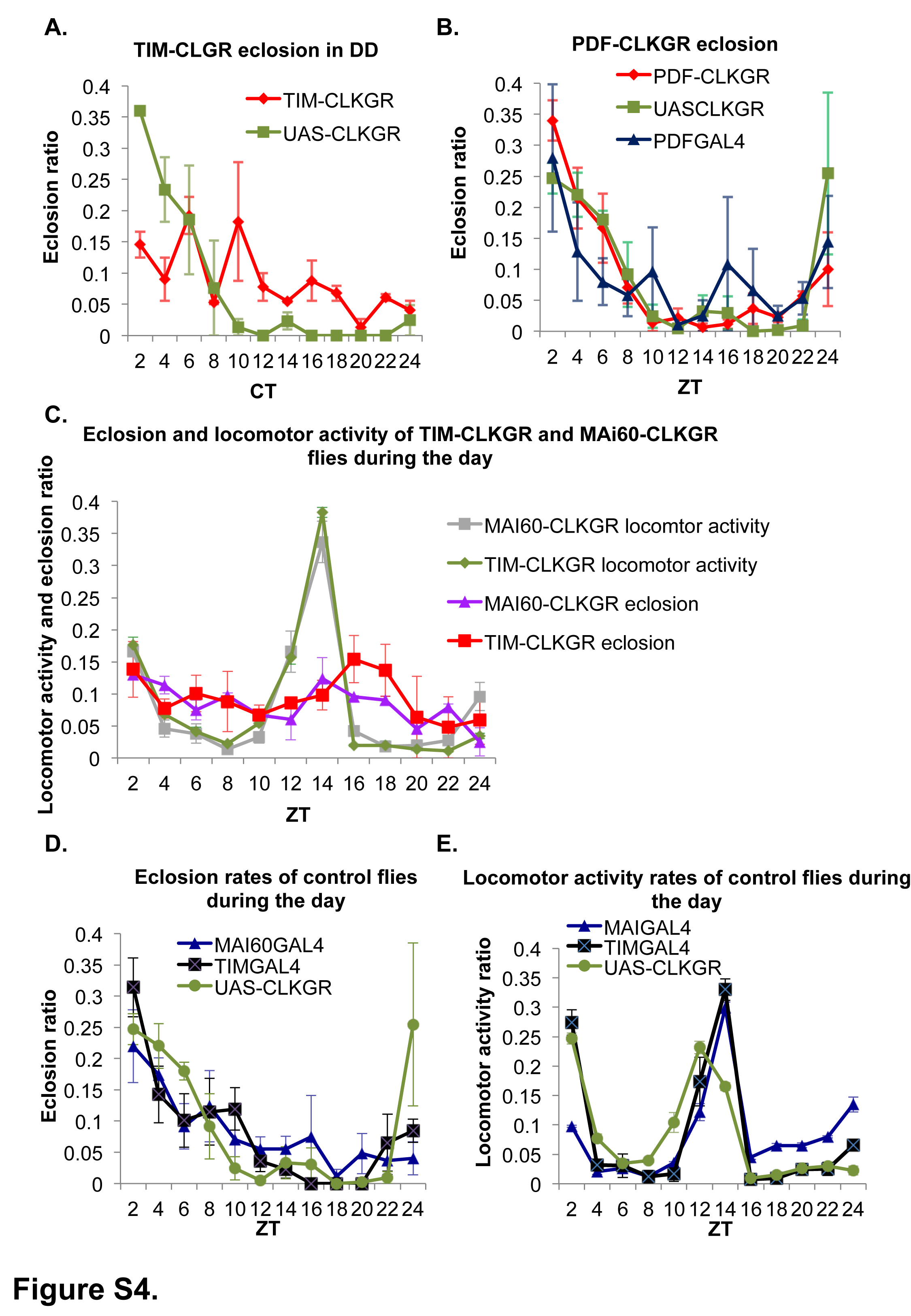

Supplement: Figure S4 — CLKGR expression impairs circadian eclosion without much effect in locomotor activity rhythms. A. Eclosion circadian gating is impaired in TIM-CLKGR flies in constant darkness conditions. We plotted the ratio between the number of the flies that emerged in two hours intervals and the total amount of flies that emerged in 24 hours for TIM-CLKGR flies and UAS-CLKGR (UAS-ClkGR/+) flies. Values are the means of 2 biological repeats (50 to 100 flies in each repeat). Error bars represent SEM. One-way Anova was performed to determine statistical significance of the differences between timepoints, for UAS-CLKGR eclosion. p<0.01. Experiment was performed during the first day in constant darkness conditions (DD1). B. Expression of CLKGR in the LNvs (PDF expressing cells) does not affect circadian eclosion rhythms. Conditions as in Figure 4A. Flies lines: PDF-CLKGR (pdf-gal4/+; UAS-ClkGR/+), PDFGAL4 (pdf-gal4/+), UAS-CLKGR (UAS-ClkGR/+). One way Anova was performed to determine statistical significance of the differences between timepoints. p<0.01. C. TIM-CLKGR and MAI60-CLKGR flies have strong locomotor activity rhythms and impaired eclosion rhythms. We measured locomotor activity during 24 hours in 12∶12 LD condition, and plot the ratio between the level of locomotor activity in 2 hours intervals and the total levels of locomotor activity in 24 hours. Values for locomotor activity are mean of 3 repeats (flies number in each repeat 27–32). Eclosion ratios are plotted as in Figure 4A. Error bars represent SEM. One-way Anova was performed to determine statistical significance of the differences between timepoints. p<0.0001 for locomotor activity and not significant for eclosion profiles. D. and E. Control flies UAS-CLKGR (UAS-ClkGR/+), TIMGAL4 (tim-gal4/+) and MAI60GAL4 (flies that carry the P{GawB}Mai60 insertion) show circadian eclosion and circadian locomotor activity ratios. Conditions as in C. One-way Anova was performed to determine statistical significance of the differe [file pgen.1004252.s005.tif]

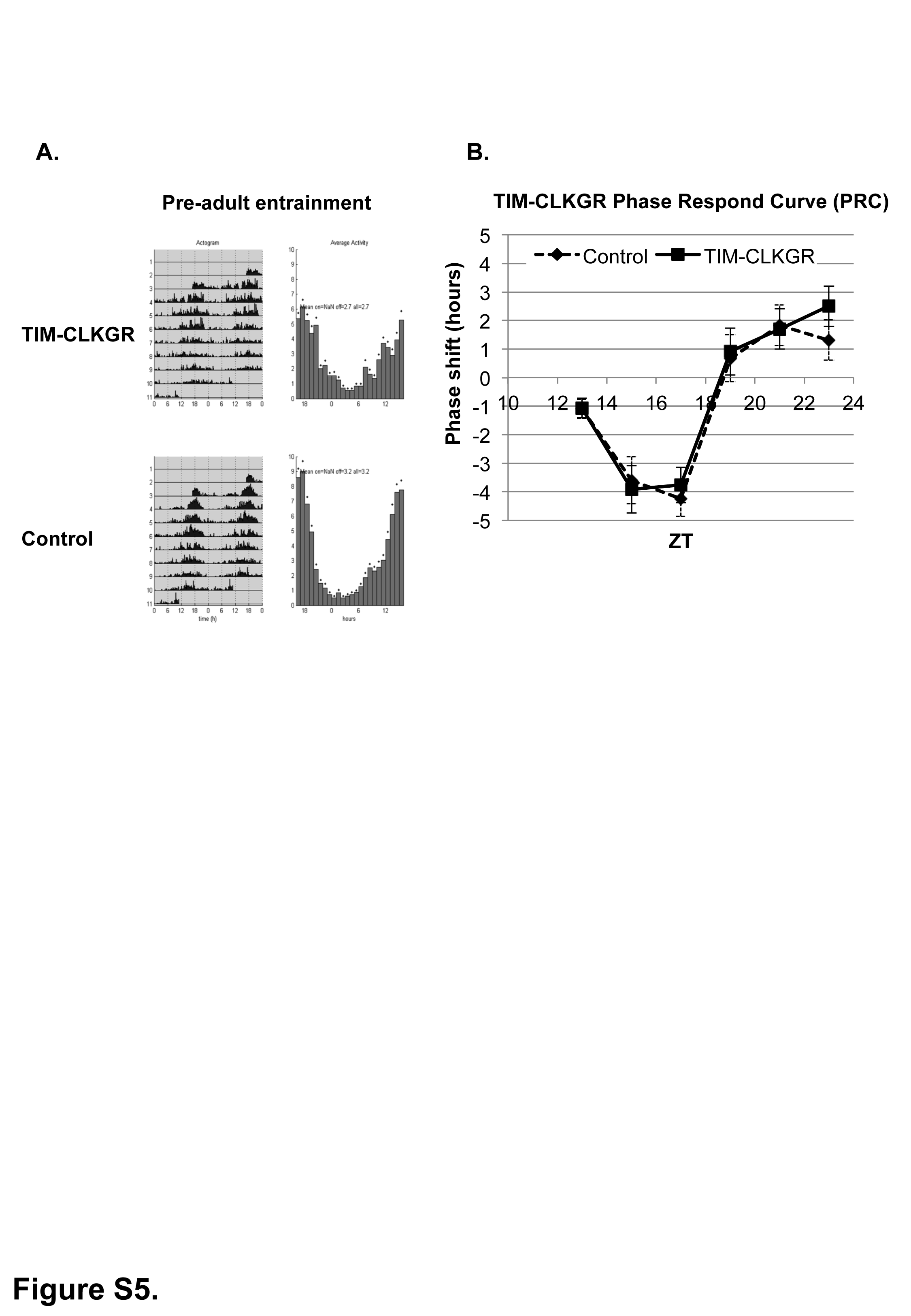

Supplement: Figure S5 — TIM-CLKGR flies have normal photoreception. A. TIM-CLKGR flies can be synchronized in the pre-adult stage. We entrained TIM-CLKGR and control flies during the larval and pupal stages to LD cycles and transferred to behavioral tubes directly in constant darkness (DD). We then assayed locomotor activity rhythms. We observed that these rhythms are synchronized with the larval/pupal entrainment light regime. Right plot flies average locomotor activity during ten days period after eclosion, left plot average activity per day. TIM-CLKGR and control (tim-gal4/+) flies were assayed. B. Phase Respond Curve (PRC) is indistinguishable between control and TIM-CLKGR flies. TIM-CLKGR phase respond curve is similar to control (tim-gal4/+). The time onset of the photic stimuli was plotted on the X-axis (ZT, in hours). The phase response was plotted on the Y-axis as the difference (in hours) from the phase of untreated flies. Mean of two repeats. Error bars represents standard deviation. (TIF) [file pgen.1004252.s006.tif]

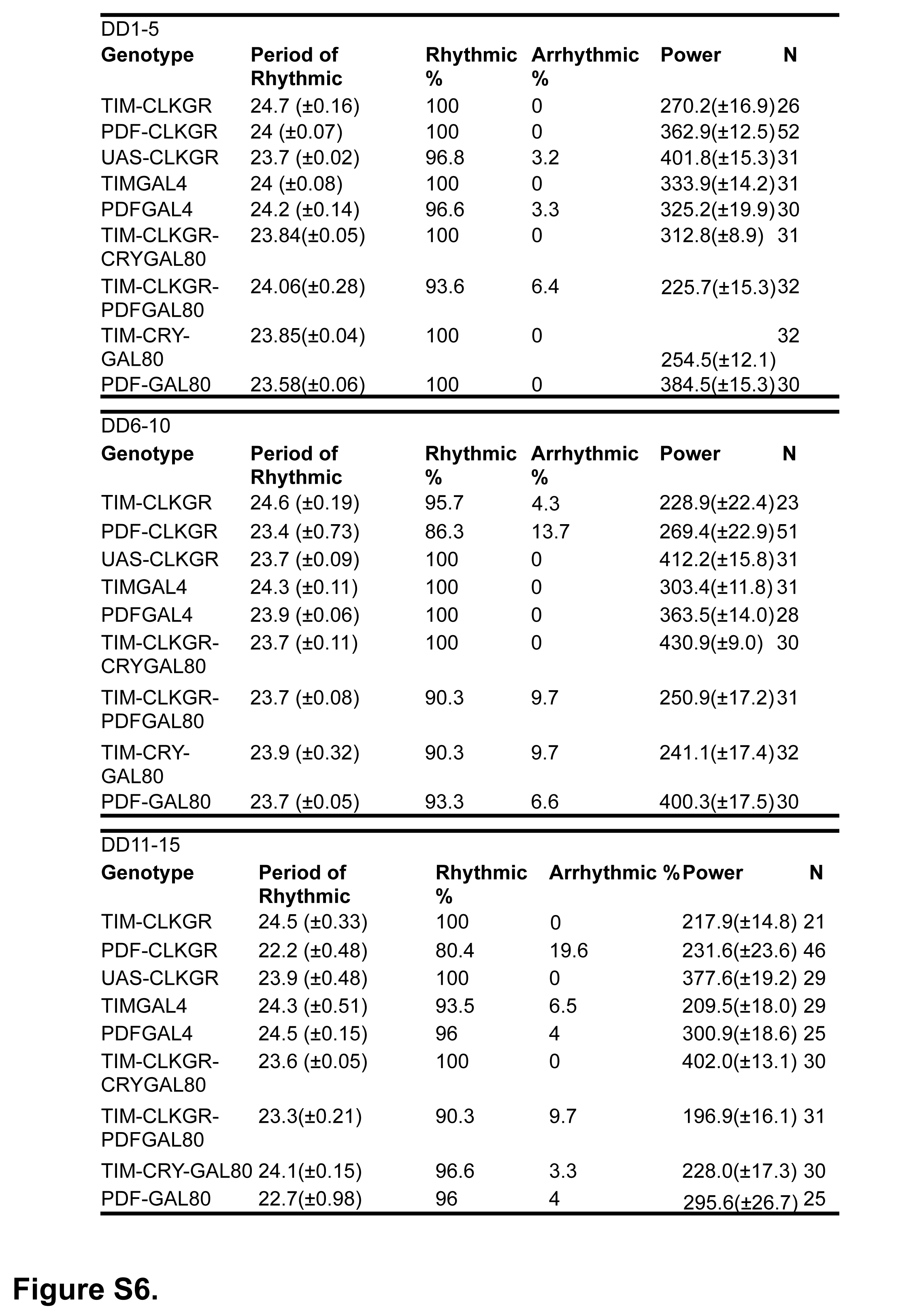

Supplement: Figure S6 — Behavioral characterization of CLKGR flies and control flies during 15 day in DD. Table sections show data for: DD 1–5, DD 6–10 and DD 11–15. Average period of rhythmic flies, rhythmic flies percentage and average power were calculated by chi square power. p<0.05. SEM is shown in brackets. Fly strains: TIM-CLKGR, PDF-CLKGR (pdf-gal4/+;UAS-ClkGR/+), UAS-CLKGR (UAS-ClkGR/+), TIMGAL4 (tim-gal4/+), PDFGAL4 (pdf-gal4/+), TIM-CLKGR-CRYGAL80 (tim-gal4/+;UAS-ClkGR,cry-gal80/+), TIM-CLKGR-PDFGAL80 (tim-gal4/+;UAS-ClkGR,pdf-gal80/+), TIM-CRY-GAL80 (tim-gal4/+;cry-gal80/+) and PDF-GAL80 (pdf-gal80). (TIF) [file pgen.1004252.s007.tif]

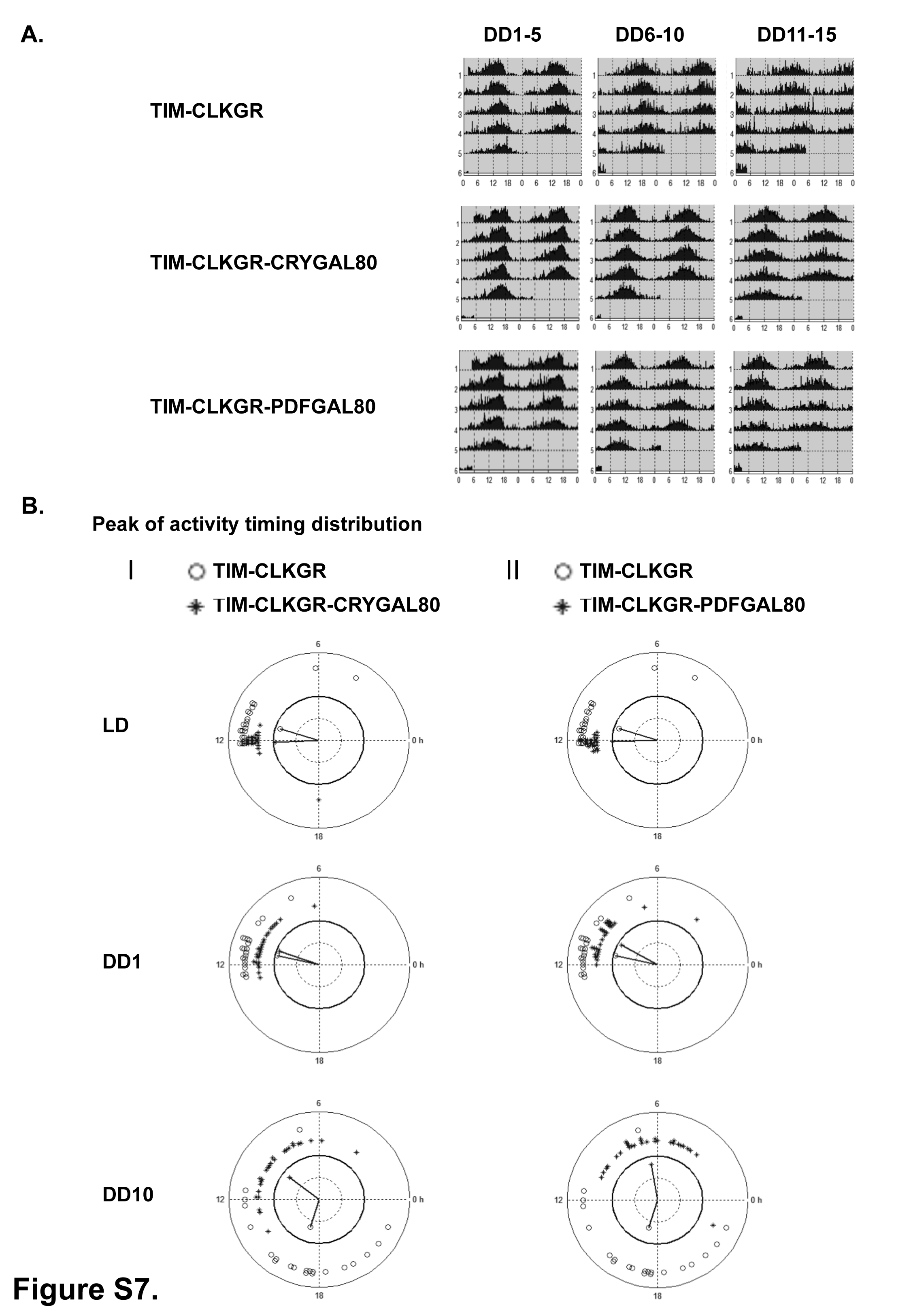

Supplement: Figure S7 — Expression of CLKGR in the TIM+CRY− or TIM+PDF− cells does not result in the behavioral defects observed in TIM-CLKGR flies. A. TIM-CLKGR flies display less robust rhythmic behavior after long time in constant darkness than TIM-CRY-GAL80 (tim-gal4/+;UAS-ClkGR,cry-gal80/+) and TIM-CLKGR-PDFGAL80 (tim-gal4;UAS-ClkGR,pdf-gal80/+). Behavior was plot for the first 5 days in constant darkness (DD1–5), days 6 to 10 in constant darkness (DD6–10) and days 11 to 15 in constant darkness (DD11–15) (rhythms data and flies numbers are shown in Figure S6). B. TIM-CLKGR flies display more spread peaks of activity after prolonged times in constant darkness than TIM-CRY-GAL80 and TIM-CLKGR-PDFGAL80 flies. Peak of activity of each fly are plotted in circular chart for the last day of 12∶12 LD conditions before transferred to constant darkness, first day in constant darkness (DD1) and day 10 in constant darkness (DD10). White circles represent TIM-CLKGR individual flies. I) Black dots represent TIM-CRY-GAL80 (tim-gal4/+;UAS-ClkGR,cry-gal80/+) individual flies. II) Black dots represent TIM-CLKGR-PDFGAL80 (tim-gal4;UAS-ClkGR,pdf-gal80/+) individual flies. (TIF) [file pgen.1004252.s008.tif]

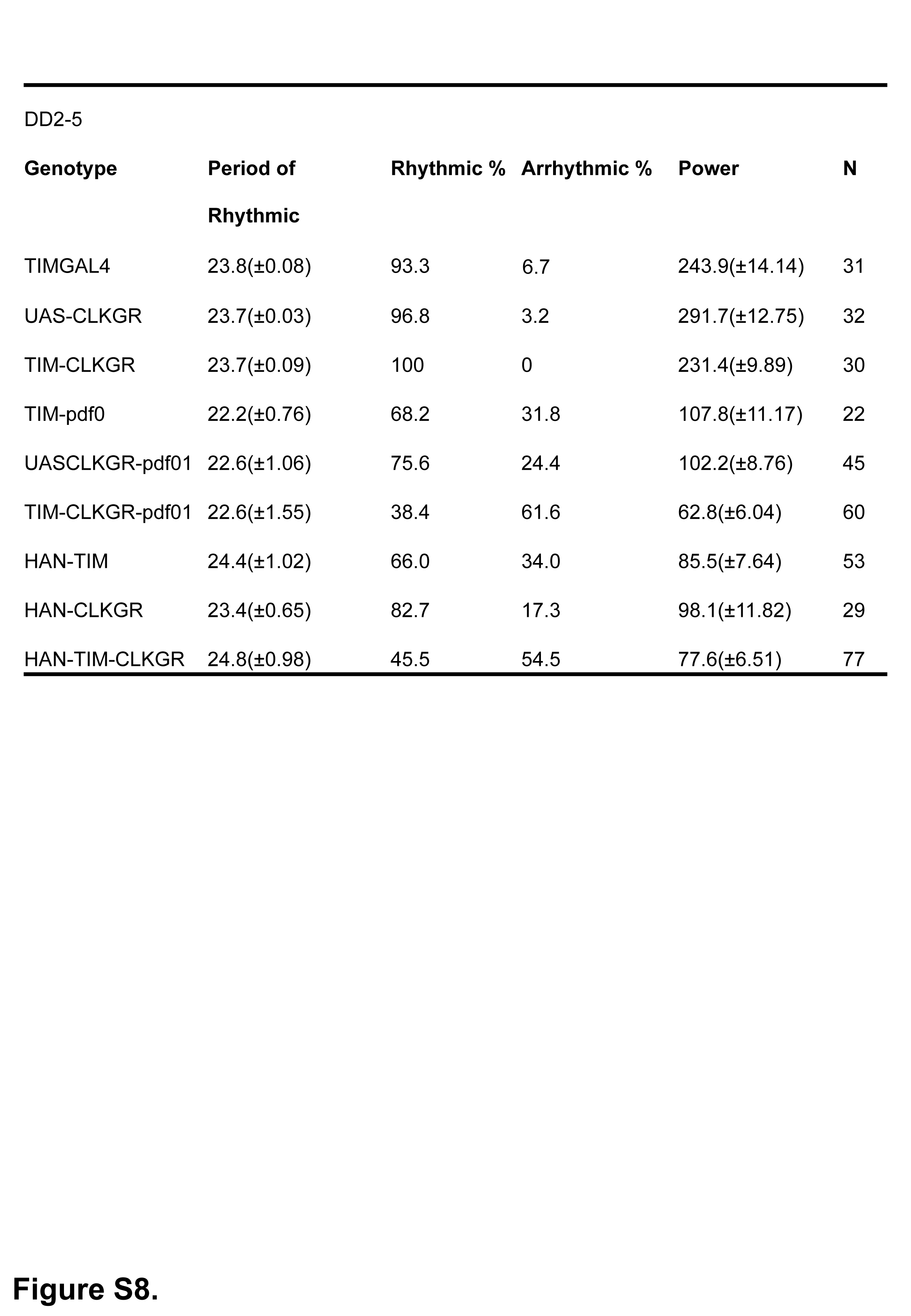

Supplement: Figure S8 — Behavioral characterization of TIM-CLKGR flies with pdf or pdfr (Han) null mutation. Rhythmicity results from day 2 to day 5 in constant darkness (DD2 to DD5). Fly strains: TIM-CLKGR, TIMGAL4 (tim-gal4/+), UAS-CLKGR (UAS-ClkGR/+), TIM-pdf01 (tim-gal4/+;pdf01), UASCLKGR-pdf01 (UAS-ClkGR/+pdf01), TIM-CLKGR-pdf01 (tim-gal4/+;UAS-ClkGR/+pdf01), HAN-TIM (han3369;;tim-gal4/+), HAN-CLKGR (han3369;;UAS-ClkGR/+), HAN-TIM-CLKGR (han3369;tim-gal4/+;UAS-ClkGR/+). Average period of rhythmic flies, rhythmic flies percentage and average power were calculated by chi square power p<0.05. SEM is shown in brackets. (TIF) [file pgen.1004252.s009.tif]

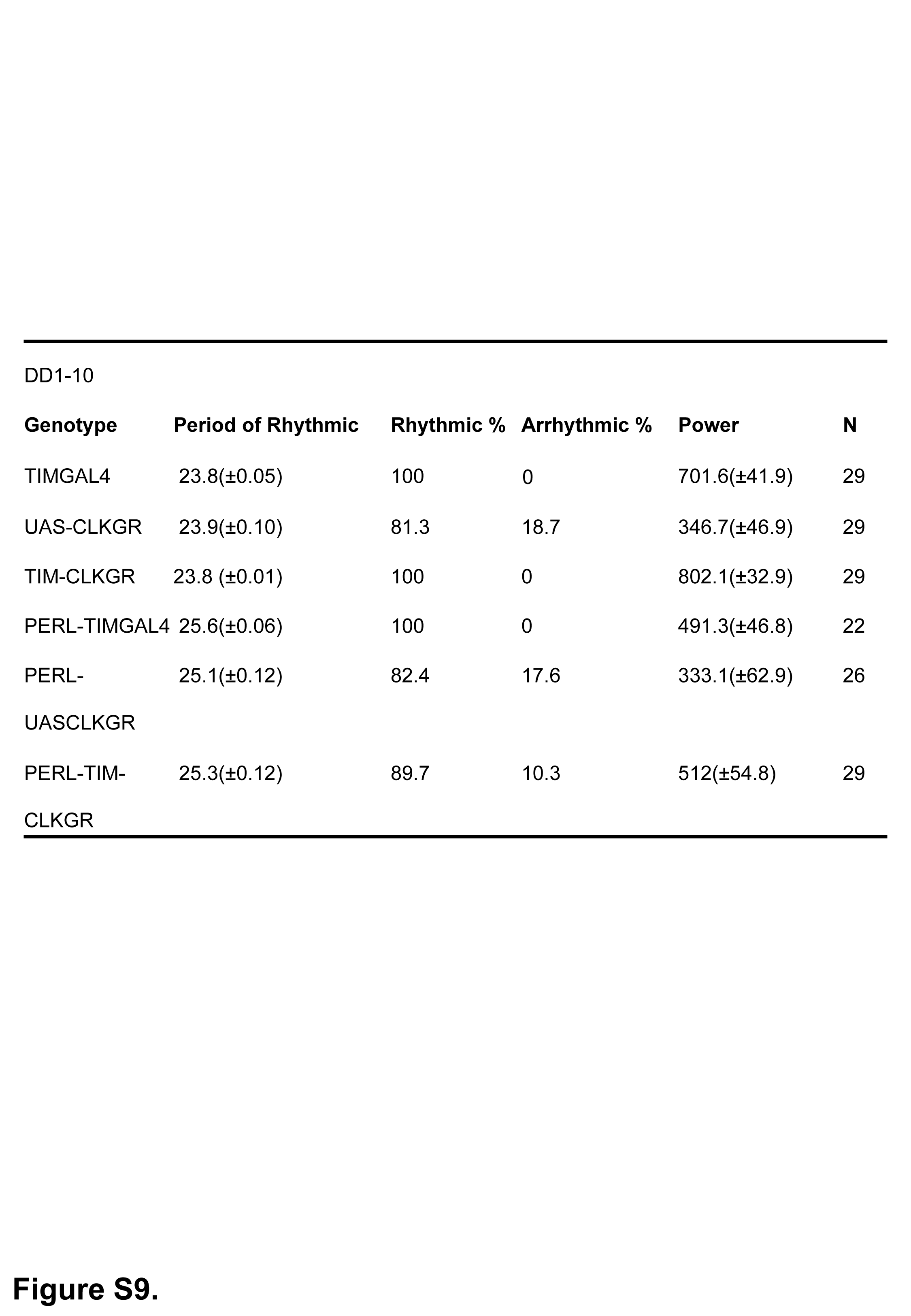

Supplement: Figure S9 — Expression of CLKGR does not genetically interact with the perL mutation. Results of locomotor activity of female flies at 10 days in constant darkness (DD1–10). TIMGAL4 (tim-gal4/+), UAS-CLKGR (UAS-ClkGR/+), TIM-CLKGR, PERL-TIMGAL4 (perL/+;tim-gal4/+), PERL-UAS-CLKGR(perL/+;;UAS-ClkGR/+), PERL-TIM-CLKGR (perL/+;tim-gal4/+;UAS-ClkGR/+). Average period of rhythmic flies, rhythmic flies percentage and average power were calculated by chi square power p<0.05. SEM is shown in brackets. (TIF) [file pgen.1004252.s010.tif]
